# Src Family Kinases Modulate the Loss of Endothelial Barrier Function in Response to TNF-α: Crosstalk with p38 Signaling

**DOI:** 10.1371/journal.pone.0161975

**Published:** 2016-09-07

**Authors:** Alejandro P. Adam, Anthony M. Lowery, Nina Martino, Hiba Alsaffar, Peter A. Vincent

**Affiliations:** 1 Department of Molecular and Cellular Physiology, Albany Medical College, Albany, New York, United States of America; 2 Department of Ophthalmology, Albany Medical College, Albany, New York, United States of America; University of Illinois at Chicago, UNITED STATES

## Abstract

Activation of Src Family Kinase (SFK) signaling is required for the increase in endothelial permeability induced by a variety of cytokines and growth factors. However, we previously demonstrated that activation of endogenous SFKs by expression of dominant negative C-terminal Src Kinase (DN-Csk) is not sufficient to decrease endothelial adherens junction integrity. Basal SFK activity has been observed in normal venular endothelia and was not associated with increased basal permeability. The basal SFK activity however was found to contribute to increased sensitivity of the venular endothelium to inflammatory mediator-induced leakage. How SFK activation achieves this is still not well understood. Here, we show that SFK activation renders human dermal microvascular endothelial cells susceptible to low doses of TNF-α. Treatment of DN-Csk-expressing cells with 50 pg/ml TNF-α induced a loss of TEER as well as drastic changes in the actin cytoskeleton and focal adhesion proteins. This synergistic effect was independent of ROCK or NF-κB activity. TNF-α-induced p38 signaling was required for the synergistic effect on barrier function, and activation of the p38 MAPK alone was also able to induce changes in permeability only in monolayers with active SFKs. These results suggest that the activation of endogenous levels of SFK renders the endothelial barrier more susceptible to low, physiologic doses of TNF-α through activation of p38 which leads to a loss of endothelial tight junctions.

## Introduction

The regulation of endothelial permeability is a critical part of the inflammatory response. During inflammation, endothelial cells (EC) must allow an increased passage of fluid, macromolecules and leukocytes to the affected tissues. However, an excessive increase in permeability may lead to systemic vascular leakage resulting in edema and a loss of organ function. Multiple signaling pathways act in coordination to ensure not only a controlled increase in permeability but also a prompt resealing of the barrier. It is well accepted that phosphorylation of tyrosines in adherens junction proteins and an increase in actin-myosin contractility are two main mechanisms leading to a quick loss of endothelial intercellular adhesions and thus an increase in vascular permeability [1–4]. In addition, mechanisms such as NF-κB-dependent transcriptional regulation are known to contribute to the loss of barrier function hours after treatment with proinflammatory cytokines such as TNF-α [5–7]. The relative importance of each pathway (cell contraction, transcriptional regulation and adherens junction phosphorylation) is still a matter of debate and the level of crosstalk between these signaling mechanisms is poorly understood.

*In vivo*, the loss of vascular barrier function can be induced by multiple factors, including VEGF, IL-6, IL-1β and TNF-α. For example, TNF-α is elevated during infection and inflammation and has been suggested to mediate the increase in vascular leakage after acute respiratory distress syndrome and sepsis [8, 9]. TNF-α plasma concentration is tightly regulated *in vivo* and is kept at very low concentrations in healthy humans. A literature survey shows that normal TNF-α plasma levels are well below 20 pg/ml [10, 11] and increase to 100–200 pg/ml during infection [12–14], only reaching 500–1000 pg/ml at the peak of severe septic shock [14]. *In vitro* studies suggesting that TNF-α can act alone to increase permeability primarily use concentrations of TNF-α ranging from 5–20 ng/ml. However, the levels of circulating cytokines are generally a poor predictor for vascular barrier loss. Clinical data have provided results suggesting that elevated levels of TNF-α are not always associated with increases in vascular permeability and, conversely, increases in vascular permeability are not necessarily associated with high plasma levels of TNF-α. For example, psoriasis is characterized by endothelial gap formation and hyperpermeability in dermal microvasculature [15, 16]. However, Arican et al [10] reported that, in psoriatic patients, TNF-α plasma concentration reached 25.7±10.62 pg/ml. Conversely, high concentrations of TNF-α as observed in some infectious diseases [11, 13] are not accompanied with widespread endothelial barrier loss. These findings would suggest that a second pathway may be needed for TNF-α-induced changes in endothelial permeability. Indeed, the ability for two mediators to contribute to changes in vascular permeability through the contribution of different signaling pathways was demonstrated by Clauss et al [17] who demonstrated that VEGF-induced increase in permeability was dependent on a transmembrane form of TNF-α.

One major signaling pathway that contributes to proinflammatory mediator–induced decreases of endothelial barrier function is the activation of Src Family Kinases (SFKs) [3, 4]. Inhibition of SFK activity prevents edema formation in animal models [18–20] and *in vitro* research demonstrated that multiple pro-edemagenic stimuli promoted the activation of SFKs and tyrosine phosphorylation of VE-cadherin [21–26]. SFK inhibitors not only inhibited the AJ proteins' phosphorylation, but also protected monolayers from increased permeability [24–27], suggesting that SFK activity plays a critical role in regulating endothelial barrier function. Although a role for SFKs in the regulation of permeability is well established, recent publications suggest a new paradigm concerning how SFKs integrate with other signaling pathways to cause mediator-induced changes in endothelial cell permeability [26, 28–31]. We have reported that activation of endogenous SFKs, by removing Csk-mediated repression of SFK activity, is not sufficient to induce a loss of barrier function in human dermal microvascular endothelial cells (HDMEC), despite a strong phosphorylation of VE-cadherin and several other SFK target proteins [26]. Moreover, we did not observe a reduction in VE-cadherin binding to either p120 or β-catenin upon SFK activation [26]. Consistent with our findings, Orsenigo et al [28] reported that venular endothelial cells show increased basal activation of Src compared to arteries, which correlated with higher levels of VE-cadherin phosphorylation at tyrosines 658 and 685, yet no change in basal permeability itself. Rather, the authors found that this difference renders the venular endothelium more susceptible to pro-edemagenic mediators. In addition, Wessel et al [29] elegantly demonstrated that vascular permeability and transendothelial migration require complex VE-cadherin phosphorylation patterns. Similarly, Sidibe et al recently showed estrous cycle-dependent VE-cadherin Y685 phosphorylation in mice, an event that was associated with normal physiology and vascular remodeling, rather than vascular leakage [30]. The same group showed that non-phosphorylatable Y685F knock-in mice display increased edema in uteri and ovaries [31]. Our previous results, together with the above-mentioned findings, strongly support a model in which other signaling molecules need to act concurrently with SFK activation in order to promote endothelial permeability. Thus, there is a clear need to fully understand the effects of SFK activation in resting and inflamed endothelia.

The experiments described in this manuscript were designed to test the hypothesis that SFK activation renders ECs susceptible to levels of TNF-α that are similar to those observed in the plasma of patients with an inflammatory response. We show that TNF-α and SFKs act synergistically to increase endothelial permeability. By using techniques that allowed us to activate individual signaling pathways independent of mediator induced receptor activation, we demonstrate that SFKs can cooperate with low levels of p38 activation, similar to those induced by low levels of TNF-α, to disrupt endothelial barrier function.

## Materials and Methods

### Cell culture

HDMEC were isolated from neonatal foreskin by incubating the obtained cells with magnetic beads coated with an antibody to CD31 (Invitrogen) as described previously [26]. HLMEC and HUVEC were obtained from Lonza. HDMEC and HLMEC were grown in EGM-2 MV (Lonza) and HUVEC were grown in EGM-2 (Lonza). Cells were used for experiments between passages 5 and 10. For all experimental protocols, cells were seeded at 10^5^ (HLMEC and HDMEC) or 4x10^4^ cells/cm^2^ (HUVEC) and incubated for 48–72 h prior to any treatment to obtain mature cell-cell junctions.

### Antibodies and reagents

TNF-α was obtained from R&D Systems. Doxycycline was from MP Biomedicals. SB203580 was from Millipore. BAY 11–7082 was from Selleck Chemicals. Y-27632, BSA, and FITC-BSA were from Sigma-Aldrich. The following antibodies were used for Western blot and/or immunofluorescence: pY419 Src (#2101), pS82 HSP27 (#2401), HSP27 (#2402) and pT180Y182 p38 (#4511) were obtained from Cell Signaling; pY118 paxillin (#ab75740) from Abcam; Src (GD11) and anti-pY (4G10) from Millipore; Csk (# sc-286), FAK (#sc-557), p38 (#sc-535) and VE-cadherin (#sc-6458) from Santa Cruz Biotechnology; paxillin (#612405) from BD Biosciences; claudin 5 (#34–1600), pY773 ITGB3 (#44-876G), pY397 FAK (#44-624G) and pY861 FAK (#44-626G) from Life Technologies; pS15 HSP27 (#2231–1) from Epitomics. Anti pT18S19 MLC was described previously [32].

### Viral Constructs

Dominant negative Csk adenovirus containing a lysine to arginine substitution at position 222 (DN-Csk) was a generous gift from Dr. S. Tanaka (Faculty of Medicine, University of Tokyo) [33]. The cDNA encoding FLAG-tagged MKK6E [34] (Addgene plasmid 13518) was cloned into pENTR11 and transferred to doxycyclin-inducible lentiviral expression vector pINDUCER22 (a generous gift from Dr. Thomas Westbrook, Baylor College of Medicine) [35] via Gateway cloning (Invitrogen). All adenoviral infections were accompanied by a control GFP and/or LacZ infection with a multiplicity of infection at or above the greatest multiplicity of infection used in the experimental groups.

### Electrical Cell-Substrate Impedance Sensor (ECIS)

Monolayer permeability was determined by measuring changes in electrical resistance using ECIS (Applied BioPhysics). Cells were seeded onto 8W10E cultureware precoated with 0.1% gelatin and incubated for 3 days. The electrical impedance across the monolayer was measured at 1 V, 4000 Hz AC and then used to calculate resistance by the manufacturer’s software. Data is presented as a plot of electrical resistance versus time.

### Albumin clearance assay

HDMEC were seeded at confluence on 0.4 μm pore transwell chambers (Corning) and incubated 48–72 h prior to the experiments. Then, cells were infected and treated as indicated in the figures. After treatments, cells were incubated in medium devoid of phenol red and containing an excess (5 mg/ml) of non-labeled albumin in both chambers. Then, 0.5 mg/ml FITC-labeled albumin was added to the upper chamber. Sixty minutes later, fluorescence in the lower chamber was measured in a multiwell fluorometer (BioTek) and compared to a FITC-albumin standard curve (0.08–40 μg/ml).

### Gel Electrophoresis and Immunoblotting

Cells grown on 6 or 12-well plates were scraped after lysis with 250 μl of Laemmli buffer containing the following protease and phosphatase inhibitors: Complete protease inhibitor mixture (Roche Applied Science), PhosSTOP phosphatase inhibitor mixture (Roche Applied Science), 0.1 M NaF and 0.1mM pervanadate (Sigma-Aldrich). After boiling, a total of 20 μl of cell lysate per lane was loaded on standard SDS-PAGE gels and transferred to nitrocellulose membranes (Bio-Rad). Immunoblots were performed by blocking the membranes with 3% nonfat dry milk or 5% BSA in PBS/Tween and incubating overnight at 4°C with respective primary antibodies. Secondary anti-mouse, anti-rabbit, or anti-goat antibodies conjugated with peroxidase (Jackson ImmunoResearch Laboratories) were incubated for 1 h at room temperature. Membranes were developed using Clarity (Bio-Rad) or SuperSignal West Pico or Femto (Pierce) chemiluminescent substrate and a LAS-3000 imaging system (Fujifilm).

### Immunofluorescence Microscopy

Immunofluorescence studies were performed by seeding 80,000 cells on 8-well μ-slide chambers (Ibidi) or 8-well glass culture slides (BD Falcon) precoated with 0.1% gelatin. Three days after seeding, cells were infected with adenovirus to express LacZ or DN-Csk, or treated as indicated. Cells were then fixed with 4% paraformaldehyde (Affymetrix) in PBS for 30 min at 4°C, washed twice with PBS, and processed for immunofluorescence at room temperature. Briefly, cells were permeabilized with 0.1% Triton X-100 (Sigma) in Tris-buffered saline (TBS-TX) for 15 min, treated with Image-IT FX signal enhancer (Invitrogen) for 30 min and then blocked with 5.5% bovine serum in TBS-TX. Antibodies (described above) were incubated for 2 h at room temperature. After washes in TBS-TX, secondary anti-goat, anti-rabbit, or anti-mouse antibodies conjugated with Alexa Fluor 488, Alexa Fluor 594, or Alexa Fluor 647 (Invitrogen) were incubated for 1 h at room temperature in the presence or absence of Alexa Fluor 488- or Alexa Fluor 594-conjugated phalloidin and 4,6-diamidino-2-phenylindole (DAPI, Invitrogen). When required, slides were mounted using Fluoromount aqueous mounting medium (Sigma-Aldrich) and #1.5 glass coverslips (Thermo Scientific). Images were obtained using a Zeiss Axio Observer Z1 inverted microscope or a Leica SPE confocal microscope and analyzed using the manufacturer’s software.

### Fluorescence quantification

Individual channels of raw Zeiss images were exported to 16-bit grayscale TIFF files without any prior adjustments to contrast or brightness. TIFF files were loaded onto ImageJ/Fiji. Pearson’s colocalization measurements were performed using the Coloc 2 plugin version 2.1.0 (http://imagej.net/Coloc_2). For nuclear fluorescence intensity measurements, the DAPI channel was used to create a binary mask that was processed for particle analysis. The individual ROI obtained from this step were then used to obtain the average fluorescent intensity for the phosphorylated p38 channel. A square ROI of the same size in each image was used for background subtraction.

### Statistical Analyses

ECIS data at single time points from multiple independent experiments were pooled for statistical analysis. Graphs are presented in S6 Fig as mean ± SEM (n = 6–12). Experiments containing multiple groups were analyzed by one-way or two-way ANOVA followed by Dunnett or Tukey post-hoc tests, respectively. In experiments with only two group comparisons, the data was analyzed using a two-tailed Student’s T-test. A p<0.05 was considered statistically significant.

## Results

**Fig 1:**
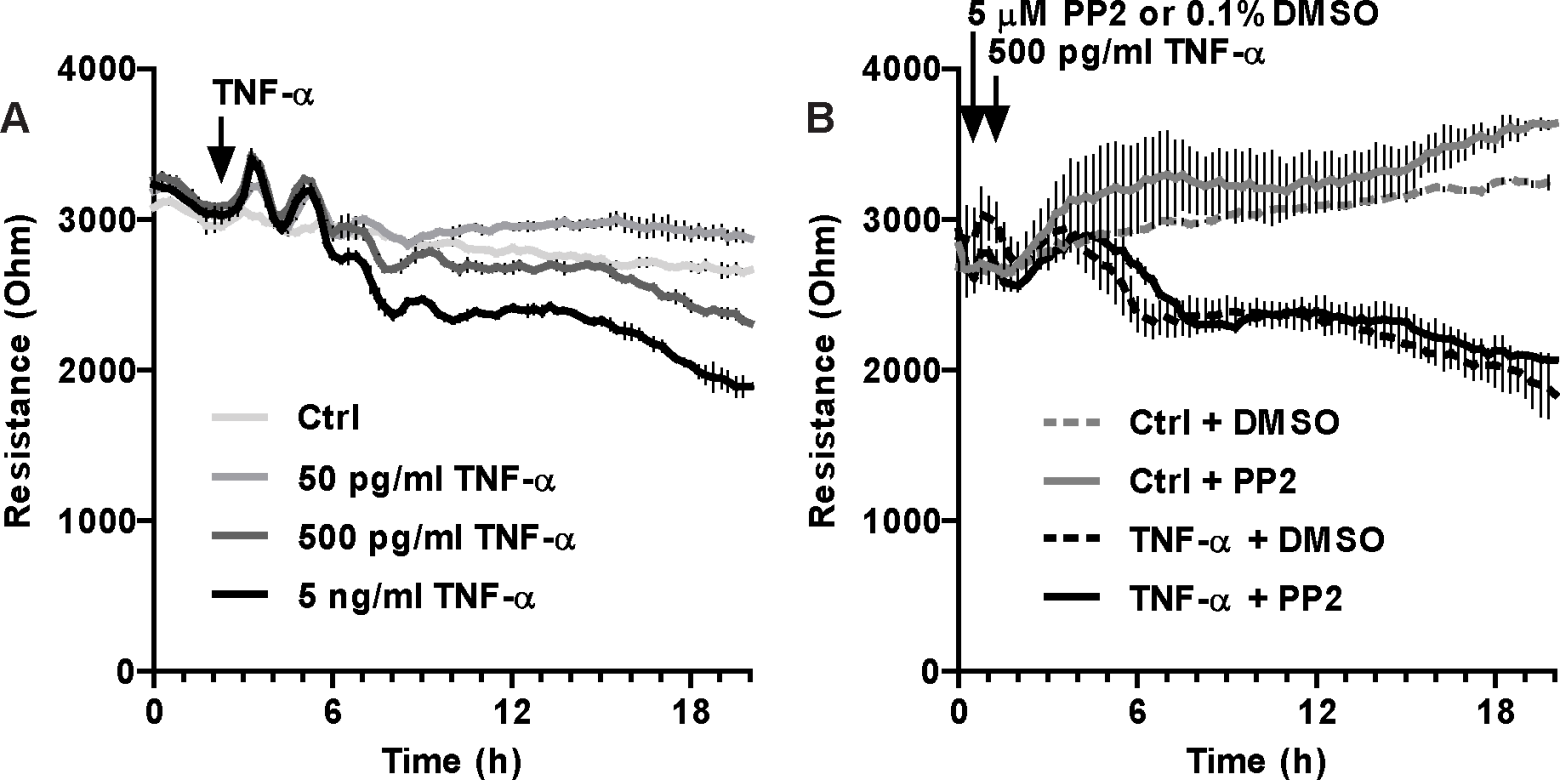
TNF-α-dependent loss of monolayer resistance in HDMEC does not depend on SFK activity. ***A***, Three days after cells were seeded at confluence on 8W10E ECIS electrodes, complete growth media was replaced by EBM-2 containing 0.3% FBS and incubated for another 16 hours. Then, cells were treated with different amounts of TNF-α (as shown). TEER at 4 kHz was measured every 5 minutes for at least 20 hours post-treatment. ***B***, Cells were treated as in A, except cells were pretreated with either DMSO (vehicle) or PP2 (SFK inhibitor) for 30 minutes prior to TNF-α addition. Notice the lack of response to TNF-α doses of 50 pg/ml or below as well as the inability of PP2 to prevent the loss of barrier function induced by a high TNF-α dose. Results are representative of at least three independent experiments performed in duplicate. Bar graphs with data presented as mean±SEM and statistical analysis of TEER for the 20 h time point are shown in S6A and S6B Fig.

**Fig 2:**
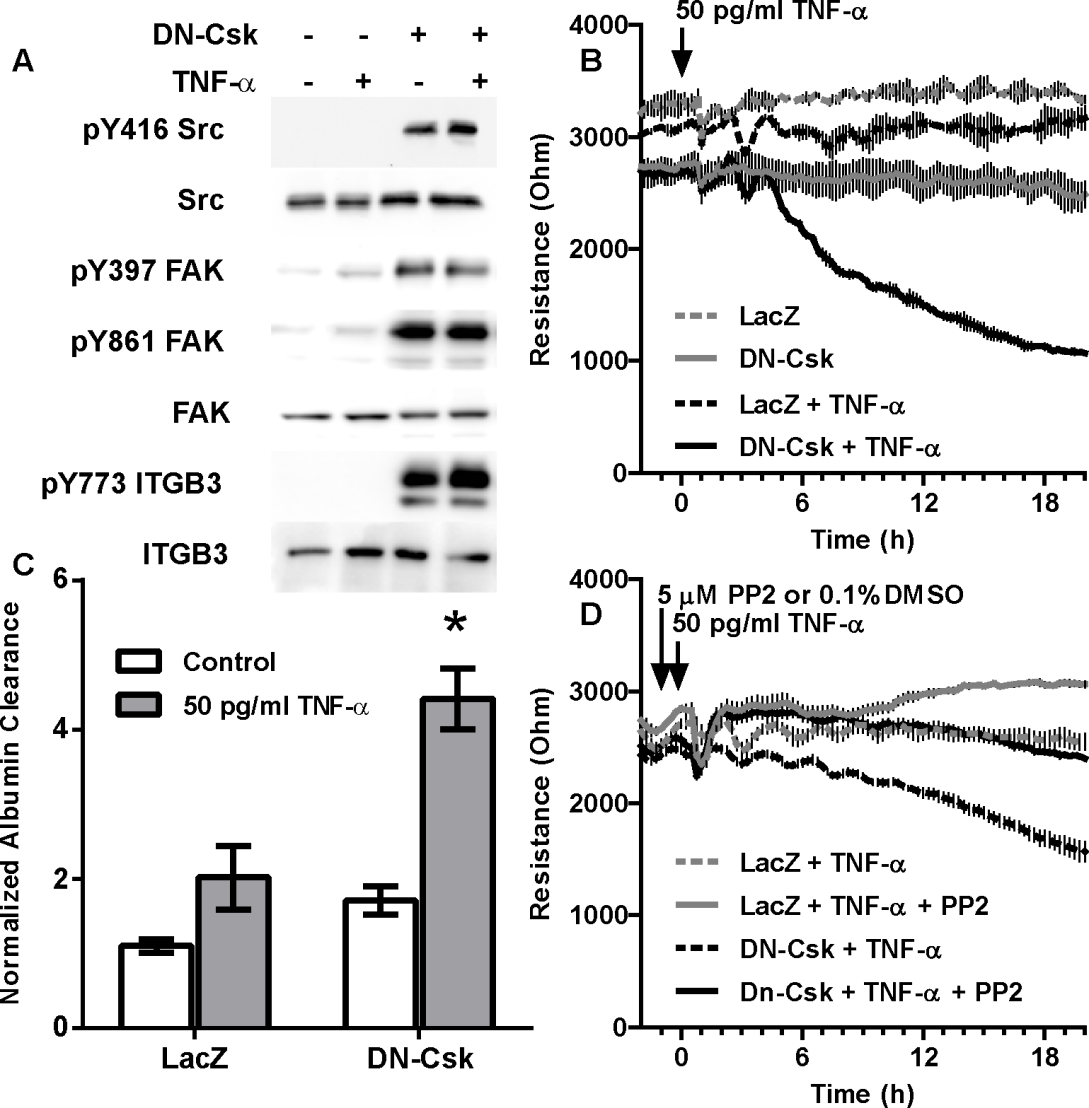
Constitutive activation of SFKs through expression of DN-Csk renders HDMEC susceptible to low doses of TNF-α. Cells were seeded at confluence and incubated for 72 hours. Then, growth media was replaced by low serum media and cells were infected with adenovirus to express either LacZ (control) or DN-Csk. After another 16 h, cells were treated with low-dose TNF-α. ***A***, Western blot analysis demonstrating the increased tyrosine phosphorylation at multiple SFK substrates. ***B***, TEER measurements on ECIS electrodes. ***C***, FITC-Albumin permeability assay on Transwell chambers. ***D***, TEER of cells pretreated with either DMSO or PP2 for 30 minutes prior to TNF-α addition. Results are representative of at least three independent experiments. Notice that the synergistic effect of DN-Csk expression concurrently with a low-dose TNF-α can be prevented by the SFK inhibitor PP2. Data presented as mean±SEM (each experiment performed in duplicate). Asterisk, p<0.05 (Two-way ANOVA and Tukey’s multiple comparison post-hoc test). Bar graphs with data presented as mean±SEM and statistical analysis of TEER for the 20 h time point corresponding to B and D are shown in S6C and S6D Fig respectively.

Initial experiments were performed to determine the effect of TNF-α treatment at and above concentrations found in the plasma of patients with diverse inflammatory and infectious diseases [10, 11, 13, 14]. We found that TNF-α treatment induced a dose-dependent loss of transendothelial electric resistance (TEER) in confluent HDMEC monolayers (Fig 1A). A dose of 500 pg/ml TNF-α induced a significant drop in TEER, with doses of 5–10 ng/ml causing maximal decrease in TEER over a 20 hour period. In contrast, a dose of 50 pg/ml TNF-α did not promote significant changes in TEER (Fig 1A) or albumin clearance (Fig 2C). Surprisingly, the loss of barrier function induced by 500 pg/ml of TNF-α was not sensitive to pharmacological SFK inhibition by PP2 (Fig 1B).

**Fig 3:**
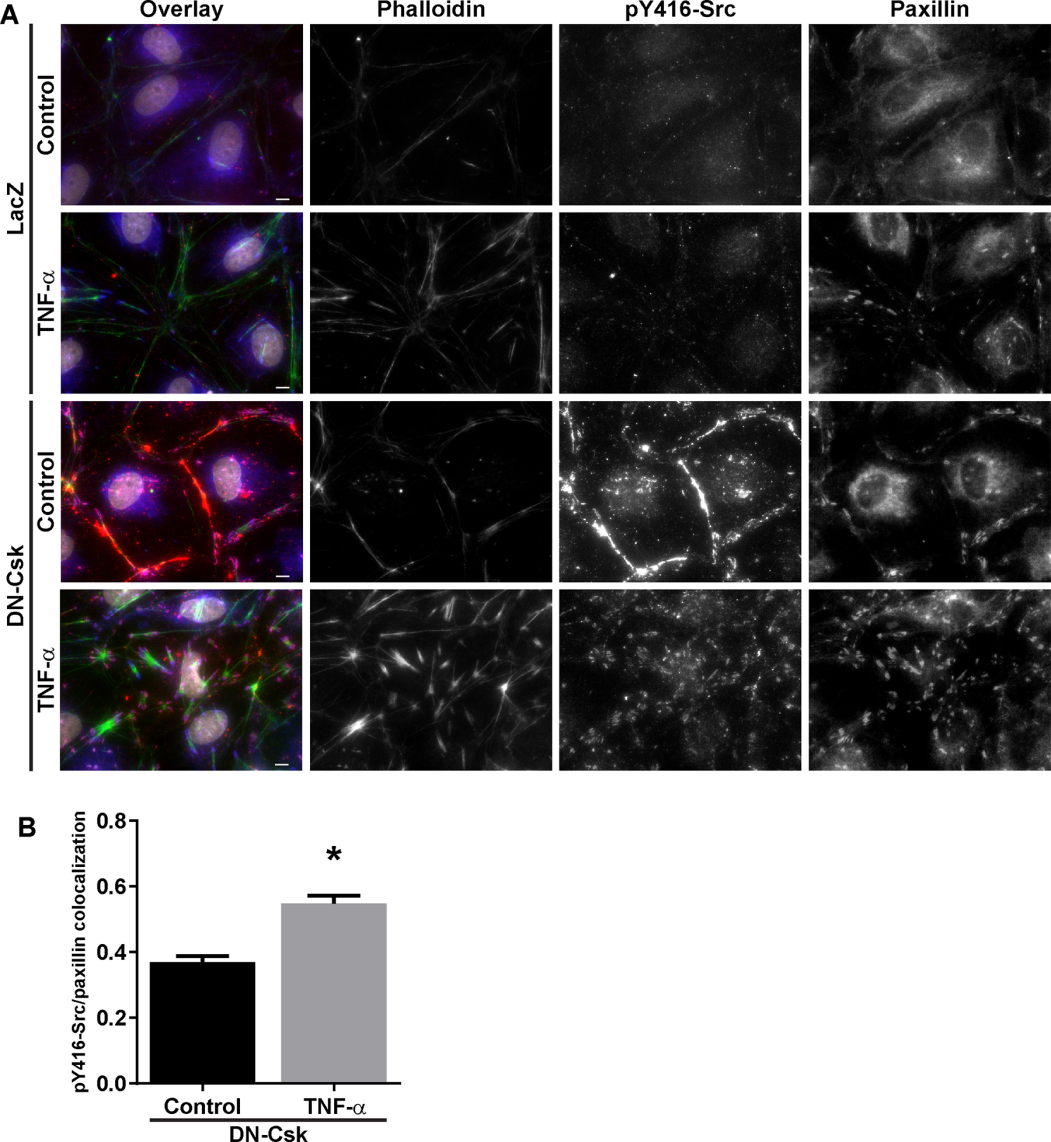
Csk inhibition alters the actin remodeling induced by TNF-α treatment. Cells were seeded at confluence and incubated for 72 hours. Then, growth media was replaced by low serum media and cells were infected with adenovirus to express either LacZ (control) or DN-Csk. After another 16 h, cells were treated with low-dose TNF-α. Cells were fixed at 6 h post TNF-α treatment. ***A***, Immunofluorescence microscopy was performed to detect pY419 Src, paxillin and F-actin (phalloidin). Nuclei were counterstained with DAPI. ***B***, Pearson’s coefficient was calculated to assess the level of colocalization between active SFK and paxillin signals in DN-Csk-expressing cells. Asterisk, p<0.05 (Student’s T test, 6 fields per condition). Note the drastic relocalization of active Src from the cell-cell junctions to cell-matrix adhesions upon dual DN-Csk and TNF-α treatments, together with a loss of cortical actin and the appearance of short and thick actin bundles. Results are representative of at least four independent experiments.

The levels of tyrosine phosphorylation in endothelial cells may vary and, at least in HUVEC, have been shown to depend on the time post-confluency [36]. We consistently observed low levels of basal SFK activity and total tyrosine phosphorylation in post-confluent HDMEC. In order to mimic the basal activation observed in venous cells, we overexpressed a dominant negative form of the SFK inhibitory kinase Csk (DN-Csk) [26, 33]. Consistent with our previous report, adenoviral delivery of DN-Csk did not induce a significant loss of endothelial resistance, despite high levels of Src activation and overall tyrosine phosphorylation (Figs 2A and 3). In some experiments, however, we observed a small increase in the permeability of HDMECs expressing DN-Csk that did not reach statistical significance (Fig 2C). We then tested whether constitutive SFK activity renders these cells more susceptible to a treatment with TNF-α. As shown in Fig 2, we observed a marked decrease in barrier function when HDMEC expressing DN-Csk were challenged with 50 pg/ml TNF-α, as measured by ECIS (Fig 2B) and protein permeability (Fig 2C). The decrease in TEER produced by low dose TNF-α (50 pg/ml) in the presence of DN-Csk (LD-TNF/DN-Csk) was completely abrogated by pretreatment with PP2, showing the importance of SFK activation to this response (Fig 2D). Interestingly, this sensitization may be unique to certain endothelial subtypes, as HDMEC, but not HLMEC or HUVEC (S1 Fig), showed an increased permeability after DN-Csk infection and low-dose TNF-α treatment. Immunofluorescent staining against VE-cadherin and ZO-1 shows the appearance of gaps in the monolayer only in cells challenged with the dual treatment (S2A and S2B Fig). Furthermore, LD-TNF/DN-Csk cells showed markedly reduced levels of claudin 5 at the cell-cell junctions (S2C Fig). This finding is consistent with recent observations by Clark et al that treatment of HDMEC with a high dose of TNF-α can promote the loss of junctional claudin 5 [6]. The low dose of TNF-α alone was unable to displace junctional claudin 5. Surprisingly, expression of DN-Csk alone promoted claudin 5 aggregation at the cell-cell borders. The significance of this behavior is unknown (S2C Fig).

**Fig 4:**
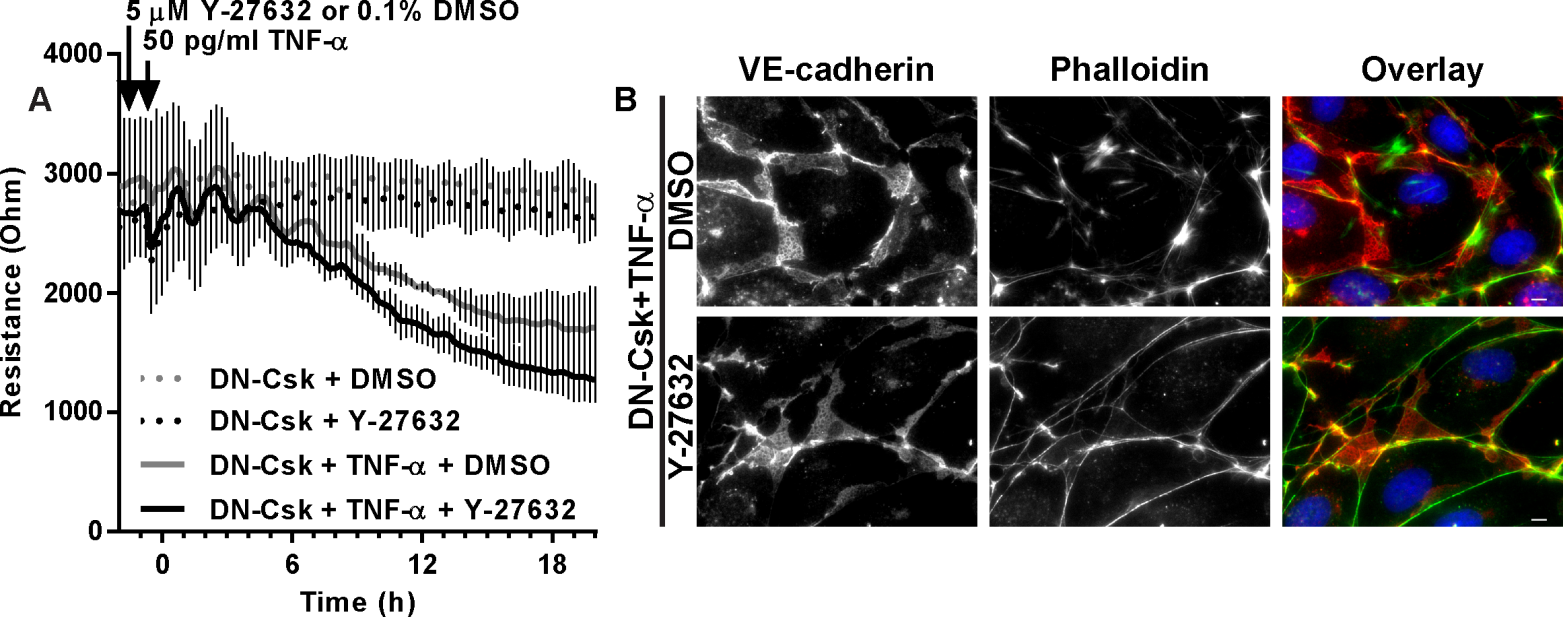
Blockade of actin stress fiber formation by ROCK inhibition does not prevent LD-TNF/DN-Csk-induced loss of barrier function. Cells were seeded at confluence and incubated for 72 hours. Then, growth media was replaced by low serum media and cells were infected with adenovirus to express either LacZ (control) or DN-Csk. After another 16 h, cells were pretreated with DMSO (vehicle) or Y-27632 (ROCK inhibitor) for 30 minutes prior to TNF-α addition. ***A***, TEER measurements on ECIS electrodes. Notice the lack of effect of Y-27632 treatment on the loss of TEER in LD-TNF/DN-Csk cells. Data presented as mean±SEM. ***B***, Cells were fixed 24 h after TNF-α treatment and IF was performed to detect VE-cadherin and F-actin (phalloidin). Nuclei were counterstained with DAPI. Notice that the formation of actin bundles that can be observed in DMSO-treated cells is absent in Y-27632-treated cells. Results are representative of at least three independent experiments performed in duplicate. Bar graph (mean±SEM) and statistical analysis of TEER for the 20 h time point corresponding to A is shown in S6E Fig.

TNF-α can promote an increase in stress fibers in endothelial cells [37, 38], and a shift in actin distribution from a peripheral actin ring to stress fibers has been associated with increased permeability in response to multiple edemagenic factors [39, 40]. High dose TNF-α-induced loss of barrier function, however, appears to be independent of actin stress fiber formation [41, 42]. To better understand how TNF-α promoted an increase in monolayer permeability in the context of activated SFK, we performed immunofluorescence stainings to determine if SFK activation was able to alter the TNF-α-induced increase in actin stress fibers. As shown in Fig 3A, TNF-α-treated HDMEC display long and thin stress fibers that span the cell body. In contrast, DN-Csk-expressing cells did not show an increase in stress fibers, but instead had a thicker peripheral actin ring that colocalized with active Src staining. Surprisingly, LD-TNF/DN-Csk cells contained thick and short actin bundles. These bundles contained large amounts of paxillin and active Src at their ends (Fig 3A). This increase in colocalization of pY416-Src and paxillin was statistically significant as determined by Pearson’s colocalization coefficient (Fig 3B). These actin bundles were also decorated with phosphorylated MLC, suggesting an active contraction (S3 Fig). Despite this finding, pharmacological inhibition of ROCK activity by pretreatment with Y27632 was unable to prevent the loss of resistance in LD-TNF/DN-Csk cells (Fig 4A), despite completely preventing the formation of the actin bundles (Fig 4B). This result thus suggests that although this dual treatment can induce a drastic rearrangement of the actin cytoskeleton, this cytoskeletal change is not required for the increased permeability.

**Fig 5:**
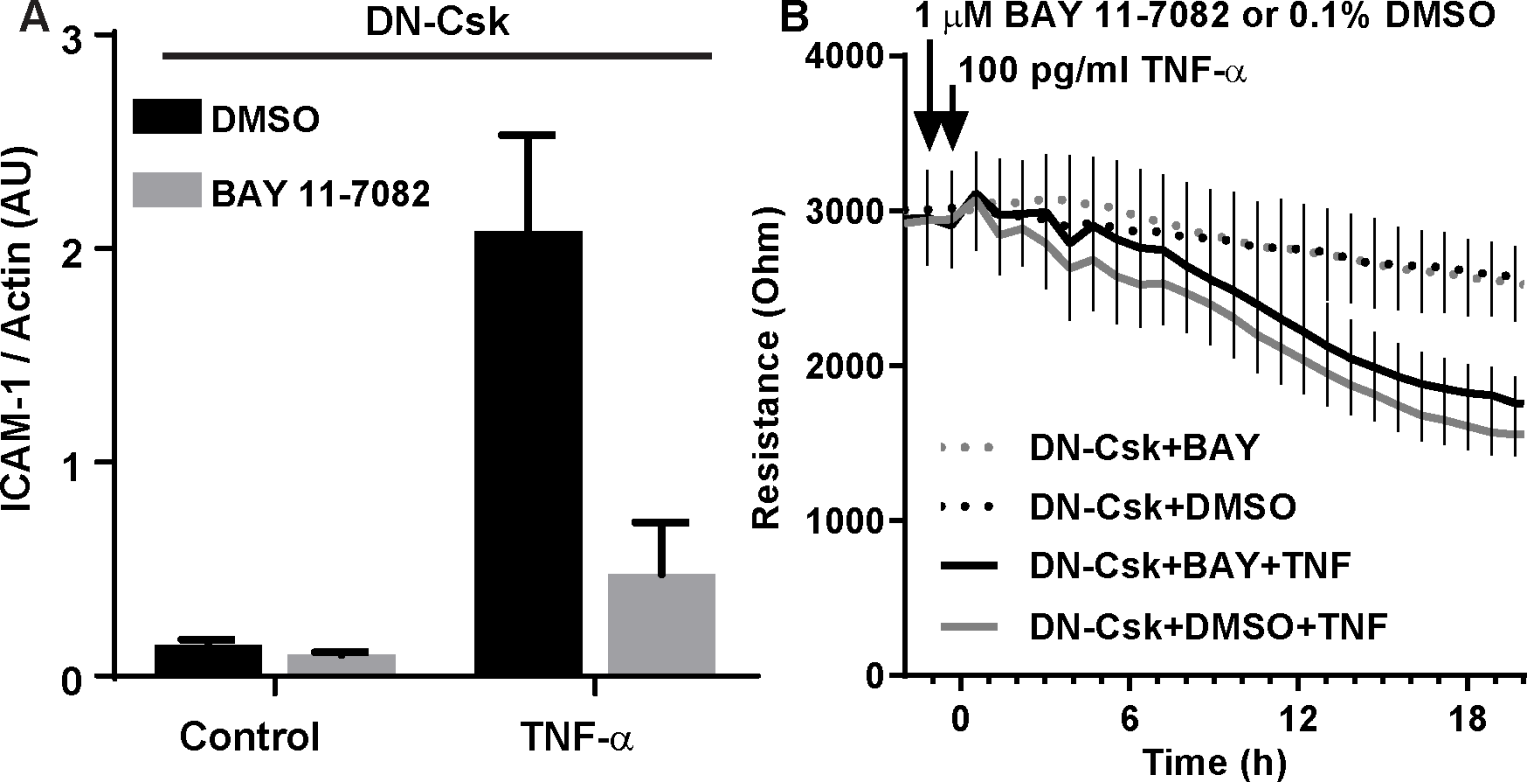
NF-κB is dispensable for the loss of barrier function in LD-TNF/DN-Csk cells. Cells were seeded at confluence and incubated for 72 hours. Then, growth media was replaced by low serum media and cells were infected with adenovirus to express either LacZ (control) or DN-Csk. After another 16 h, cells were pretreated with DMSO (vehicle) or BAY 11–7082 (NF-kB inhibitor) for 30 minutes prior to TNF-α addition. ***A***, Quantification of three independent Western blot experiments demonstrating that BAY 11–7082 is able to prevent ICAM-1 expression in LD-TNF/DN-Csk cells after 8 h of treatment with TNF-α. ***B***, TEER measurements on ECIS electrodes. Notice the lack of effect of BAY 11–7082 treatment on the loss of TEER in LD-TNF/DN-Csk cells. Results are representative of three independent experiments performed in duplicate. Data presented as mean±SEM. Bar graph (mean±SEM) and statistical analysis of TEER for the 20 h time point corresponding to B is shown in S6F Fig.

The transcription factor family NF-κB has also been shown to mediate TNF-α-induced endothelial barrier breakdown, possibly through a loss of junctional claudin 5 [5–7]. We thus sought to test whether NF-κB activity was responsible for the loss of barrier function in LD-TNF/DN-Csk cells. Pretreatment of these cells with 1 μM BAY 11–7082 abrogated TNF-α-induced increase in ICAM-1, a well-known NF-κB effector gene in endothelial cells (Fig 5A). Despite this inhibition, BAY 11–7082 pretreatment was unable to prevent the increase in permeability in LD-TNF/DN-Csk cells (Fig 5B), allowing us to exclude NF-κB activation as the mechanism downstream.

**Fig 6:**
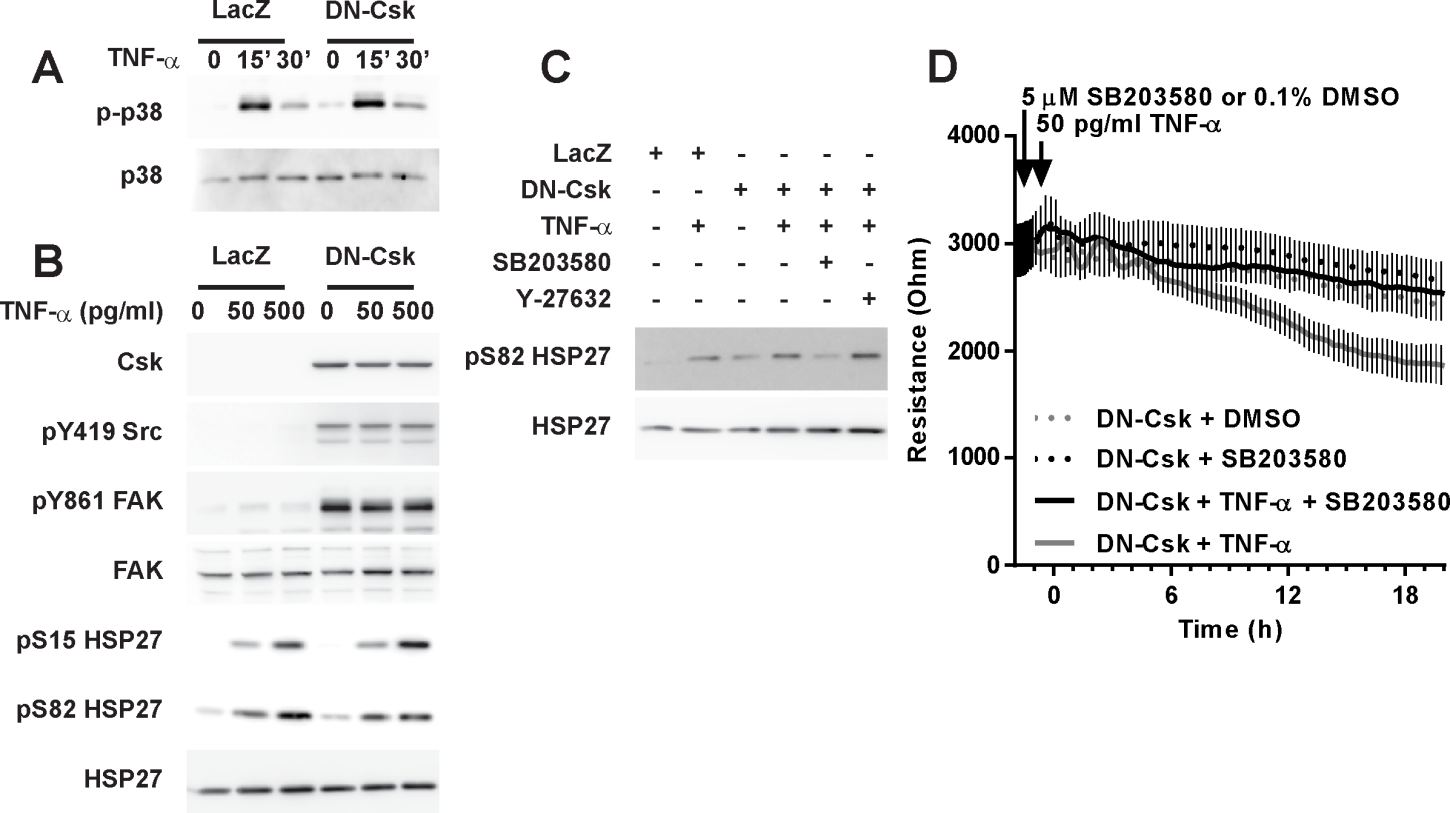
p38 activity is required for the increased permeability in LD-TNF/DN-Csk cells. Cells were seeded at confluence and incubated for 72 hours. Then, growth media was replaced by low serum media and cells were infected with adenovirus to express either LacZ (control) or DN-Csk. ***A***, 16 h later, cells were treated with low dose TNF-α for 15 or 30 minutes and assayed for p38 phosphorylation by Western blot. ***B***, Cells were treated with low or high dose TNF-α and assayed for p38 and SFK activity 30 minutes post-treatment (as measured by pY-Src, pY-FAK and pS-HSP27) by Western blot. ***C***, Cells were pretreated for 30 minutes with either DMSO (vehicle), SB203580 (p38 inhibitor) or Y-27632 (ROCK inhibitor), treated as in A, and assayed for pS82-HSP27 by Western blot. ***D***, Cells were pretreated with either DMSO or SB203580 30 minutes prior to TNF-α addition and then TEER was measured on ECIS electrodes for an additional 20 hours. Data presented as mean±SEM. Notice that p38 inhibition reversed the loss of barrier function in LD-TNF/DN-Csk cells. Results are representative of at least three independent experiments (n = 2–4 per experiment). Bar graph and statistical analysis of TEER for the 20 h time point corresponding to C is shown in S6G Fig.

P38MAPK may also mediate edemagenic signaling in endothelial cells [17, 32, 43, 44]. Thus, we sought to determine whether p38 activation mediated the loss of resistance in LD-TNF/DN-Csk cells. As shown in Fig 6A and 6B, TNF-α treatment induced the phosphorylation of both p38 and its downstream effector HSP27. This activation was independent of the status of SFK activity. Moreover, inhibition of p38 activity by pretreatment with SB203580 abrogated the increase in permeability in LD-TNF/DN-Csk cells (Fig 6C and 6D), demonstrating that p38 kinase activity is required for this effect.

**Fig 7:**
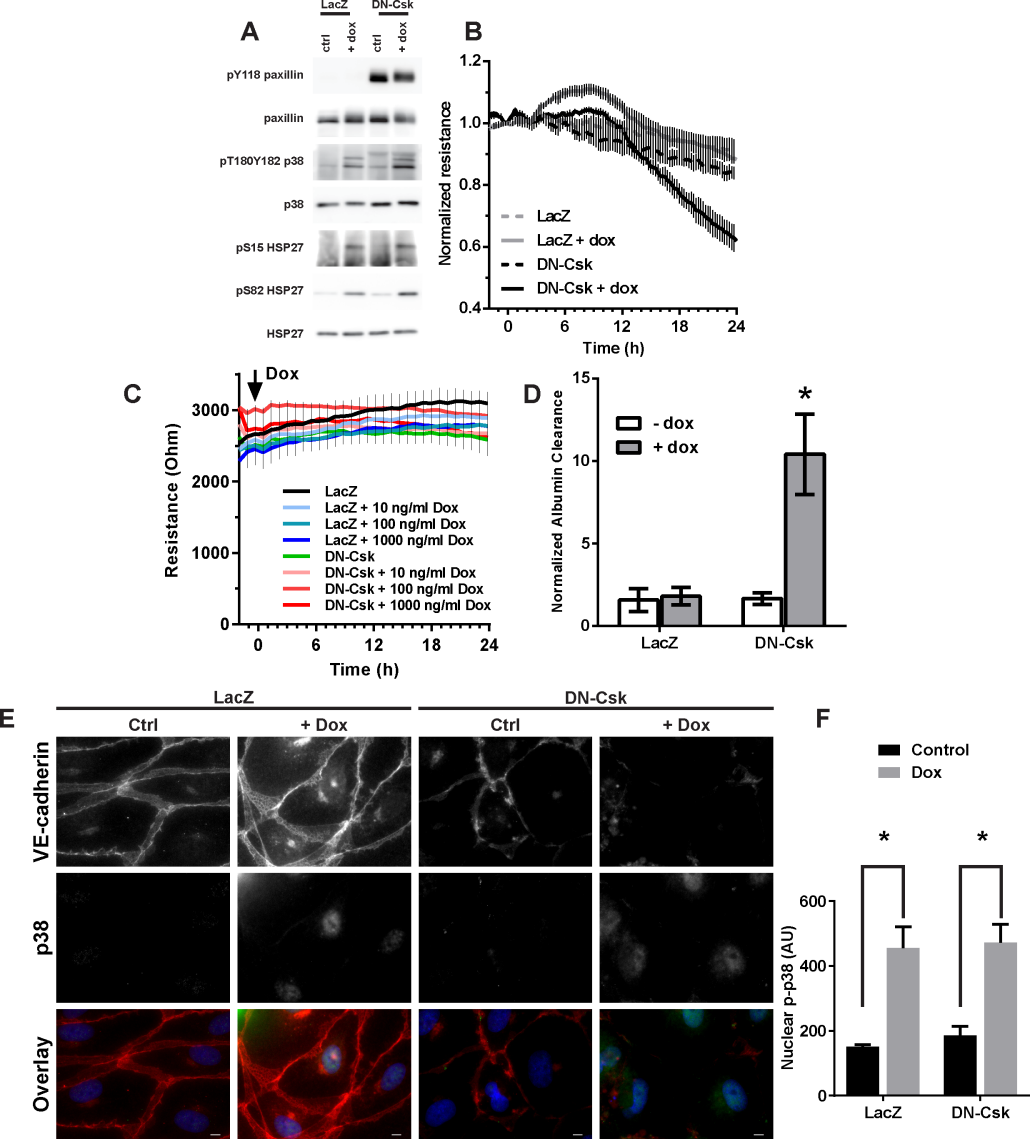
Concurrent activation of p38 and SFK pathways is sufficient to promote an increase in monolayer permeability. Pre-confluent monolayers of HDMEC were infected with lentivirus containing active FLAG-tagged MKK6 construct (iMKK6E) under the control of an inducible promoter. These cells were reseeded and allowed to become confluent for 3 days. Then, confluent iMKK6E-HDMEC monolayers were infected with adenovirus to express either LacZ or DN-Csk. After an additional incubation of 24 h, cells were treated with or without doxycycline to induce the expression of MKK6E in low serum media. ***A***, Cells were then lysed 18 h later and assayed for SFK and p38 signaling by Western blot, demonstrating independent pathway activation. ***B***, TEER was measured on ECIS electrodes in cells treated as in A. ***C***, Cells expressing either LacZ or DN-Csk were treated with different concentrations of doxycycline and TEER was measured on ECIS electrodes for 24 h. ***D***, FITC-Albumin permeability assay on Transwell chambers 18 h after doxycycline addition. Data presented as mean±SEM. Asterisk, p<0.05 (Two-way ANOVA and Tukey’s multiple comparison post-hoc test). Note the synergistic action to promote an increase in endothelial permeability. Results are representative of at least three independent experiments. ***E***, Cells were fixed 18 h after doxycycline addition and stained for phosphorylated (pT180Y182) p38 and VE-cadherin. Nuclei were counterstained with DAPI. Note the nuclear localization of phospho-p38 in cells treated with doxycycline. Results are representative of at least three independent experiments performed in duplicate. ***F***, Nuclear (pT180Y182) p38 fluorescence intensity was quantified from three fields from each treatment. Bar graphs and statistical analyses of TEER for the 20 h time point corresponding to B and C are shown in S6H and S6I Fig.

The results shown above suggest that p38 activity is required for the loss of barrier function. To test whether activation of this kinase is sufficient to promote a loss of barrier function, we generated lentivirus coding a doxycycline-inducible, FLAG-tagged MKK6E construct (iMKK6E). MKK6 is a kinase upstream of p38 and expression of this phosphomimetic mutant promotes constitutive p38 activation [34]. Addition of doxycycline to iMKK6E cells rapidly induced MKK6E expression (S4 Fig) and p38 activation (Fig 7A, 7E and 7F). Doxycyclin-mediated expression of this construct, however, was not able to promote an increase in permeability of HDMEC (Fig 7B and 7D), thus suggesting that, similarly to SFK activation, p38 activity is required, but not sufficient to induce a loss of barrier function. We then used this construct to interrogate whether concurrent p38 and SFK activation were then sufficient to promote a permeability increase. As shown in Fig 7B and 7D, this dual activation promoted a drastic loss of monolayer resistance in the absence of any external edemagenic stimulus. This was due to the expression of the iMKK6E construct, as addition of doxycycline to cells not infected with the lentivirus did not affect endothelial resistance even at a dose ten times higher (Fig 7C). The p38 pathway is known to promote cytoskeletal changes through the MAPKAP2-dependent phosphorylation of HSP27 [45, 46]. Consistent with a lack of involvement of the actin cytoskeleton, however, the dual p38/SFK activation promoted the formation of monolayer gaps and a marked loss of junctional VE-cadherin (Fig 7E and S5A Fig) but did not induce a loss of peripheral actin or an increase in stress fibers (S5B Fig).

## Discussion

Although it is well accepted that endothelial SFK activation plays an important role in the regulation of vascular permeability, the mechanisms that ultimately lead to the loss of barrier function are still under investigation [3, 4]. Activation of SFKs is required for VEGF-induced permeability both *in vitro* and *in vivo* [18–21, 25, 26], but we previously showed that activation of endogenous SFKs alone is not sufficient to induce a loss of barrier function [26]. The finding that SFK-mediated tyrosine phosphorylation is required, but not sufficient, to increase endothelial monolayer permeability implies that other signaling pathways work in conjunction with SFK activation to disrupt endothelial monolayer integrity. Thus, we hypothesized that SFK activity may act by modulating the response to other edemagenic signals. In fact, Orsenigo et al showed that venules that responded to a bradykinin challenge had higher basal SFK activity and VE-cadherin phosphorylation levels [28]. Moreover, the authors showed that SFK inhibition prevented the increase in vascular permeability after bradykinin treatment. However, the vessels with increased SFK activation did not show basal leakiness, demonstrating that SFK activation *in vivo* is also insufficient to promote leakiness. Based on Western blot and immunofluorescence data, HDMEC show low levels of SFK activation *in vitro* under non-stimulated conditions. In this manuscript, we increased basal SFK activity using DN-CSK in these cells to interrogate *in vitro* whether crosstalk between SFK and TNF-α signaling mediated an increase in endothelial permeability.

We show that SFK activation in HDMEC renders these cells susceptible to a treatment with 50 pg/ml TNF-α. This dose was unable to promote a loss of monolayer resistance in the absence of SFK activation. Importantly, this is a dose that is within the range of circulating concentrations of TNF-α in patients with diverse inflammatory diseases [10, 11, 13, 14]. Understanding how other signaling can modulate the endothelial response to these low levels of TNF-α may inform us about critical pathophysiological mechanisms during inflammation. The higher basal levels of SFK activation in venular endothelial cells may explain at least in part why post-capillary venules are particularly sensitive to edemagenic stimuli. Proinflammatory factors such as TNF-α activate multiple pathways, making it difficult to assess the relative importance of each. Using a combination of viral construct delivery and pharmacological treatment, we showed that SFK can act synergistically with p38MAPK to promote an increase in vascular permeability. Neither inhibition of ROCK signaling or NF-κB transcriptional activity were able to prevent the loss of monolayer integrity in LD-TNF/DN-Csk cells. Both pathways have been shown to mediate edemagenic factor-induced barrier function loss. In contrast to other stimuli, ROCK/MLC-dependent actin reorganization has been shown to be dispensable for TNF-α-induced increase in endothelial permeability [41, 42]. We found this to be true also in the context of constitutive SFK activation. NF-κB inhibition, on the other hand, prevented TNF-α-induced loss of barrier function in endothelial cells [5–7]. Our results thus support the notion that signaling pathway crosstalk may drastically affect the mechanisms downstream of TNF-α.

As mentioned previously, the circulating levels of TNF-α in the plasma of many patients with inflammatory conditions do not exceed 200 pg/ml [10–14]. Some vascular beds, such as the pulmonary endothelium, may be exposed to much higher local concentrations during acute respiratory distress syndrome [47]. We found that, at least in HDMEC, the signaling mediators required for the increased endothelial permeability in the context of SFK activation and low TNF-α concentrations differ from those required by high levels of TNF-α alone. Our results differ from those obtained by Angelini et al [24], who reported that TNF-α treatment of endothelial cells induced tyrosine phosphorylation and that PP2, an inhibitor of Src activity, inhibited the increase in permeability produced by 100 U/ml TNF-α (equivalent to the 5 ng/ml dose used here). Surprisingly, we did not detect any changes in SFK activation after TNF-α addition. This discrepancy could be attributed to the fact that these authors added vanadate, a tyrosine phosphatase inhibitor, to the cells 20 minutes prior to collecting lysates. This would suggest that the endogenous phosphatases could maintain low levels of tyrosine phosphorylation despite an increase in kinase activity produced by TNF-α. Heterogeneity of endothelial cells may also explain the discrepancy, as we observed differences between dermal microvascular cells and lung microvascular cells or umbilical vein cells. Angelini et al [24] tested SFK dependency on human pulmonary artery endothelial cells. In fact, Piegeler et al recently showed that high dose TNF-α can promote Src activation in human lung microvascular endothelial cells [48, 49], further suggesting that the endothelial response to TNF-α may depend on the organ of origin of the cell cultures.

The identity of the main SFK member(s) responsible for the observed mechanism remains unknown. SFK members can exert complex, overlapping and even opposing activities. Except for their “unique region” near the amino terminus, the sequence and structure of the 8 SFK members are highly conserved (reviewed in [50]). This high homology is in part responsible for the ample redundancy in SFK members activation and downstream signaling [50]. This is highlighted by the seminal discoveries that Src, Fyn or Yes deficiency have only modest phenotypes [51–53], several double knockouts die perinatally or have stronger phenotypes [53–56] and a triple knockout (“SYF” mice) is embryonically lethal [57]. SFK activation and inactivation is tightly controlled by positive and negative phosphorylation events at Y419 and Y530 (human Src numbering), respectively [50, 58]. c-Src terminal kinase (Csk) is the main kinase known to promote SFK inactivation by phosphorylating Y530 [reviewed by Okada [59]]. In contrast to the redundancy observed among SFK members, Csk is present as a single gene in all animal phyla [59] and global Csk deficiency promotes constitutive SFK activation and is embryonically lethal [60, 61]. Conditional Csk knockout strategies successfully activated SFK specifically in cells from lymphocytic [62, 63], epidermal [64, 65] and neural crest [66] origin. To our knowledge, there are no published reports of endothelial-specific SFK or Csk knockouts and little is known about the relative contributions of each SFK member in endothelial permeability. Of note, Src and Yes have been shown to promote a loss of barrier function in some models [67, 68] but not others [24], while Lyn appears to be required to sustain the endothelial barrier [69] and Fyn has been shown to promote both increased permeability [68] as well as barrier function recovery [70].

The molecular targets downstream of the synergistic action of p38 and SFKs in HDMEC are still unknown. While our results suggest that the actin cytoskeleton rearrangement observed is not required for the increase in permeability, we cannot exclude the possibility of other, more subtle changes mediating the barrier breakdown. Alternatively, SFK activity may modulate the transcriptional response to an inflammatory stimulus, ultimately leading to barrier loss. Recently, Clark et al showed that TNF-α induces a biphasic response in endothelial cells [6]. NF-κB-mediated transcriptional activity was required for the prolonged increase in monolayer permeability in this model. We observed a very similar response in TNF-α-treated HDMEC. After treatment, the cells displayed a variable resistance for the first 4–5 hours and a marked decrease immediately following this phase. It is tempting to speculate that this second phase in LD-TNF/DN-Csk cells also requires transcriptional activity. In contrast to the observations by Clark et al, who examined the mechanisms of higher doses of TNF-α, LD-TNF/DN-Csk-induced increase in permeability was not dependent on NF-κB, suggesting that other transcription factor(s) may be required downstream of this synergistic stimulus. In either case, TNF-α-mediated loss of barrier function was associated with a relocalization of VE-cadherin and claudin 5 away from the cell-cell junctions. Loss of junctional claudin 5 was apparent even in regions with normal levels of junctional VE-cadherin, so it is tempting to hypothesize that the claudin 5 loss might be an earlier event. Direct activation of p38 in the context of DN-Csk expression appears to promote a more dramatic loss of junctional VE-cadherin than the observed loss in LD-TNF/DN-Csk cells, suggesting that the MKK6E construct may not mimic perfectly TNF-α signaling in the presence of activated SFKs. Any potential differences in downstream signaling remain to be determined.

Signaling crosstalk between SFK activation and inflammatory receptor stimulation may allow endothelial cells to selectively respond to edemagenic stimuli. A two-hit model can enable the fine spatial and temporal specificity of vascular permeability. Receptor signaling modulation may also explain the hyperpermeability and endothelial gap formation in dermal microvasculature in psoriatic patients [15, 16] even with TNF-αplasma concentrations typically below 50 pg/ml [10]. Understanding the complexity and diversity of the signaling pathways regulating vascular permeability in different inflammatory conditions may allow us to better prevent and manage vascular leakage and the ensuing organ damage.

## Supporting Information

S1 Fig DN-Csk does not increase the sensitivity of HLMEC or HUVEC to low dose TNF-α. ***A*,** HLMEC were seeded at confluence and incubated for 72 hours. Then, growth media was replaced by low serum media and cells were infected with adenovirus to express either LacZ (control) or DN-Csk. After another 16 h, cells were treated with low-dose TNF-α. TEER was measured on ECIS electrodes for 24 h. ***B*,** HUVEC were seeded at confluence and incubated for 24 hours. Then, growth media was replaced by low serum media and cells were infected with adenovirus to express either LacZ (control) or DN-Csk. After another 16 h, cells were treated with low-dose TNF-α. TEER was measured on ECIS electrodes for 24 h. ***C*, *D*,** Resistance values at 20 h post-treatment were compared by 2-way ANOVA. Data in this figure comprise raw intensity values of three independent experiments performed in duplicate for each cell type.
(PDF)

S2 Fig The increase in permeability in LD-TNF/DN-Csk cells correlates with the formation of gaps in the monolayer. Cells were seeded at confluence and incubated for 72 hours. Then, growth media was replaced by low serum media and cells were infected with adenovirus to express either LacZ (control) or DN-Csk. After another 16 h, cells were treated with low-dose TNF-α. Cells were fixed at 24 h post TNF treatment. ***A*,** Immunofluorescence microscopy was performed to detect VE-cadherin and ZO-1. Nuclei were counterstained with DAPI. Note the formation of monolayer gaps with reduced presence of ZO-1 and VE-cadherin in LD-TNF/DN-Csk cells (arrows). ***B***, Fluorescence intensity profiles of linear ROI as shown in A. Notice the lack of junctional ZO-1 signal in LD-TNF/ DN-Csk cells. VE-cadherin signal intensity varied with location within the cell-cell junction but is lost at gap sites. ***C***, Immunofluorescence and confocal microscopy to detect VE-cadherin and claudin 5. Nuclei were counterstained with DAPI. Arrows mark the sites of monolayer gaps and arrowheads mark regions of claudin 5 loss without any obvious loss of junctional VE-cadherin or gap formation. Results are representative of at least three independent experiments.
(PDF)

S3 Fig Actin bundles in LD-TNF/DN-Csk cells are decorated by phosphorylated MLC. Cells were seeded at confluence and incubated for 72 hours. Then, growth media was replaced by low serum media and cells were infected with adenovirus to express either LacZ (control) or DN-Csk. After another 16 h, cells were treated with low-dose TNF-α. Cells were fixed at 24 h post TNF treatment and immunofluorescence microscopy was performed to detect phosphorylated (pT18pS19) myosin light chain and F-actin (phalloidin). Nuclei were counterstained with DAPI. Note the strong colocalization of F-actin bundles and phosphorylated MLC in LD-TNF/DN-Csk cells. Results are representative of at least three independent experiments.
(PDF)

S4 Fig FLAG-tagged MKK6E expression in Dox-treated iMKK6E cells. ***A*,** Pre-confluent monolayers of HDMEC were infected with lentivirus containing active FLAG-tagged MKK6 construct (iMKK6E) under the control of an inducible promoter. These cells were reseeded and allowed to become confluent for 3 days. Then, confluent iMKK6E-HDMEC monolayers were treated with varying concentrations of doxycycline to induce the expression of MKK6E in low serum media. Cells were lysed 24 h after doxycycline addition and blotted to detect the FLAG tag, as well as phosphorylated and total p38 and HSP27. ***B*,** Cells were infected with 0.1 μl/ml lentivirus and cultured as in ***A***. Then, confluent iMKK6E-HDMEC monolayers were infected with adenovirus to express either LacZ or DN-Csk. After additional 24 h of incubation, cells were treated with or without doxycycline to induce the expression of MKK6E in low serum media at different time points and lysed 24 h later. Shown are blots to detect FLAG tag, Csk and phosphorylated and total p38.
(PDF)

S5 Fig Dual activation of p38 and SFK pathways promotes monolayer gaps but does not lead to radial stress fiber formation. Pre-confluent monolayers of HDMEC were infected with lentivirus containing active FLAG-tagged MKK6 construct (iMKK6E) under the control of an inducible promoter. These cells were reseeded and allowed to become confluent for 3 days. Then, confluent iMKK6E-HDMEC monolayers were infected with adenovirus to express either LacZ or DN-Csk. After an additional incubation of 24 h, cells were treated with or without doxycycline to induce the expression of MKK6E in low serum media. Cells were fixed 18 h after doxycycline addition and stained for VE-cadherin (A) or active (pY416) Src and F-actin (phalloidin) (B). Nuclei were counterstained with DAPI. Note the lack of radial stress fibers or actin bundles in all four conditions. Results are representative of three independent experiments. Bars: 50 μm (A) and 5 μm (B).
(PDF)

S6 Fig Statistical analysis of ECIS data at 20 h post-treatment. Data presented as mean±SEM of at least three independent experiments combined. Panels ***A-H*** correspond to Figs 1A, 1B, 2B, 2D, 4A, 5B, 6C and 7C respectively. ***A***, One-way ANOVA with Dunnett post-hoc comparison against control. ***B***, Unpaired T test of TNF-α-treated cells in the presence of either DMSO or PP2. ***C-H***, Two-way ANOVA and Tukey’s multiple comparison post-hoc test. Asterisks denote p<0.05, while n.s. denote a non-significant change (p>0.05).
(PDF)
