# Supplementary figures and images for: Src Family Kinases Modulate the Loss of Endothelial Barrier Function in Response to TNF-α: Crosstalk with p38 Signaling
Source: PLoS One. 2016 Sep 7;11(9):e0161975. doi: 10.1371/journal.pone.0161975 (PMC5014308; doi:10.1371/journal.pone.0161975)

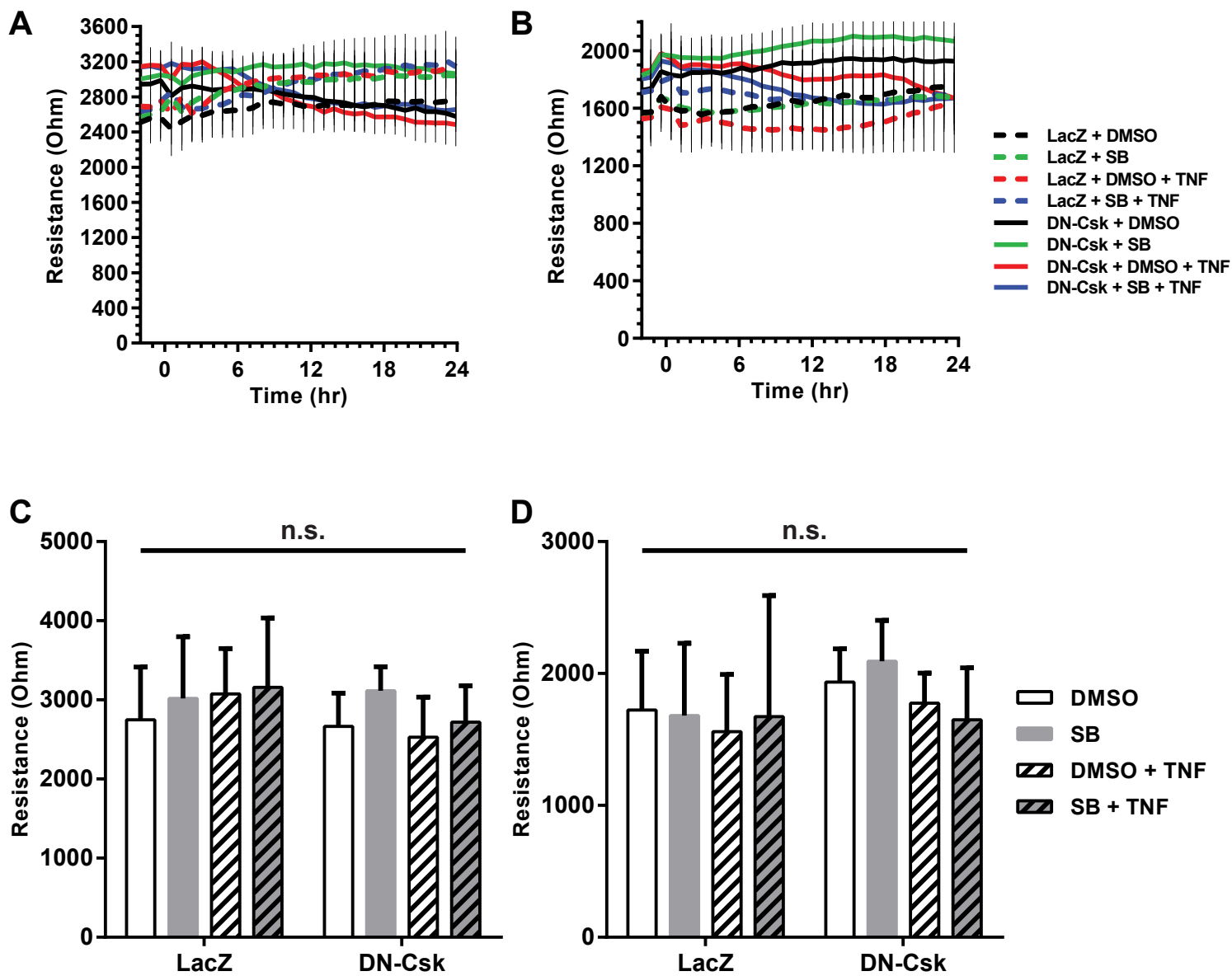

Supplement: S1 Fig — A, HLMEC were seeded at confluence and incubated for 72 hours. Then, growth media was replaced by low serum media and cells were infected with adenovirus to express either LacZ (control) or DN-Csk. After another 16 h, cells were treated with low-dose TNF-α. TEER was measured on ECIS electrodes for 24 h. B, HUVEC were seeded at confluence and incubated for 24 hours. Then, growth media was replaced by low serum media and cells were infected with adenovirus to express either LacZ (control) or DN-Csk. After another 16 h, cells were treated with low-dose TNF-α. TEER was measured on ECIS electrodes for 24 h. C, D, Resistance values at 20 h post-treatment were compared by 2-way ANOVA. Data in this figure comprise raw intensity values of three independent experiments performed in duplicate for each cell type. (PDF) [file pone.0161975.s001.pdf]

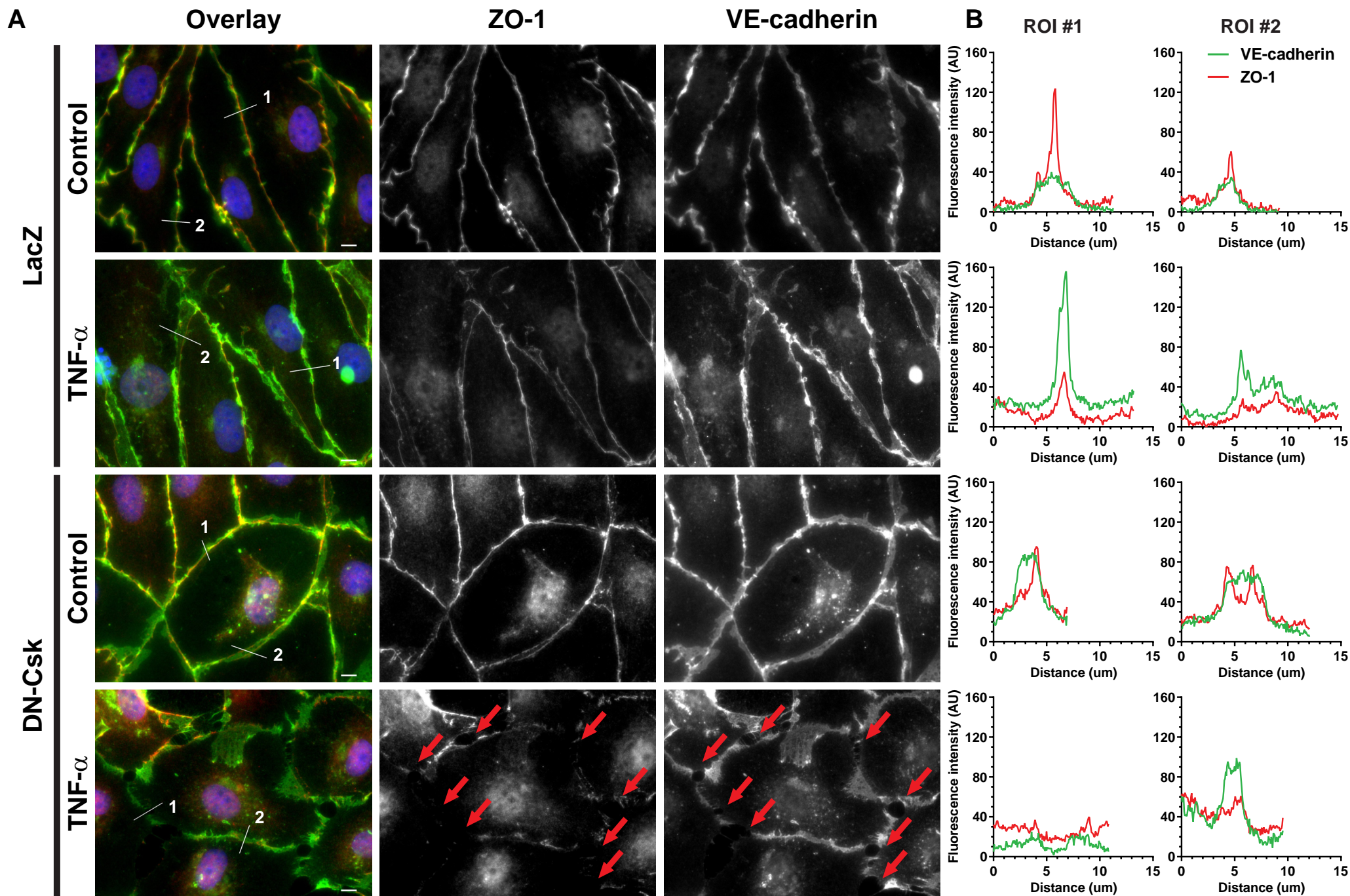

**S2 Fig**

**C**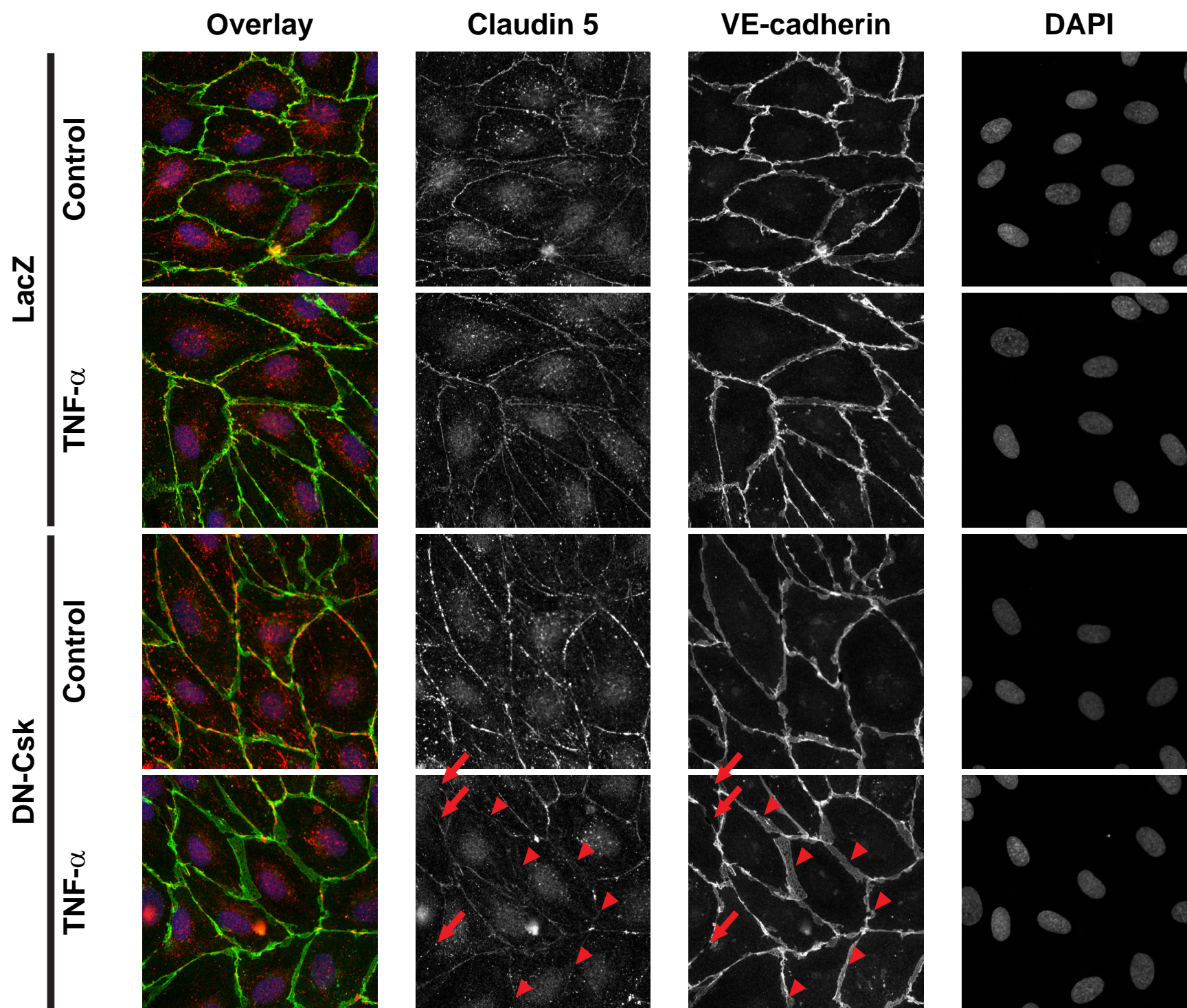

Supplement: S2 Fig — Cells were seeded at confluence and incubated for 72 hours. Then, growth media was replaced by low serum media and cells were infected with adenovirus to express either LacZ (control) or DN-Csk. After another 16 h, cells were treated with low-dose TNF-α. Cells were fixed at 24 h post TNF treatment. A, Immunofluorescence microscopy was performed to detect VE-cadherin and ZO-1. Nuclei were counterstained with DAPI. Note the formation of monolayer gaps with reduced presence of ZO-1 and VE-cadherin in LD-TNF/DN-Csk cells (arrows). B, Fluorescence intensity profiles of linear ROI as shown in A. Notice the lack of junctional ZO-1 signal in LD-TNF/ DN-Csk cells. VE-cadherin signal intensity varied with location within the cell-cell junction but is lost at gap sites. C, Immunofluorescence and confocal microscopy to detect VE-cadherin and claudin 5. Nuclei were counterstained with DAPI. Arrows mark the sites of monolayer gaps and arrowheads mark regions of claudin 5 loss without any obvious loss of junctional VE-cadherin or gap formation. Results are representative of at least three independent experiments. (PDF) [file pone.0161975.s002.pdf]

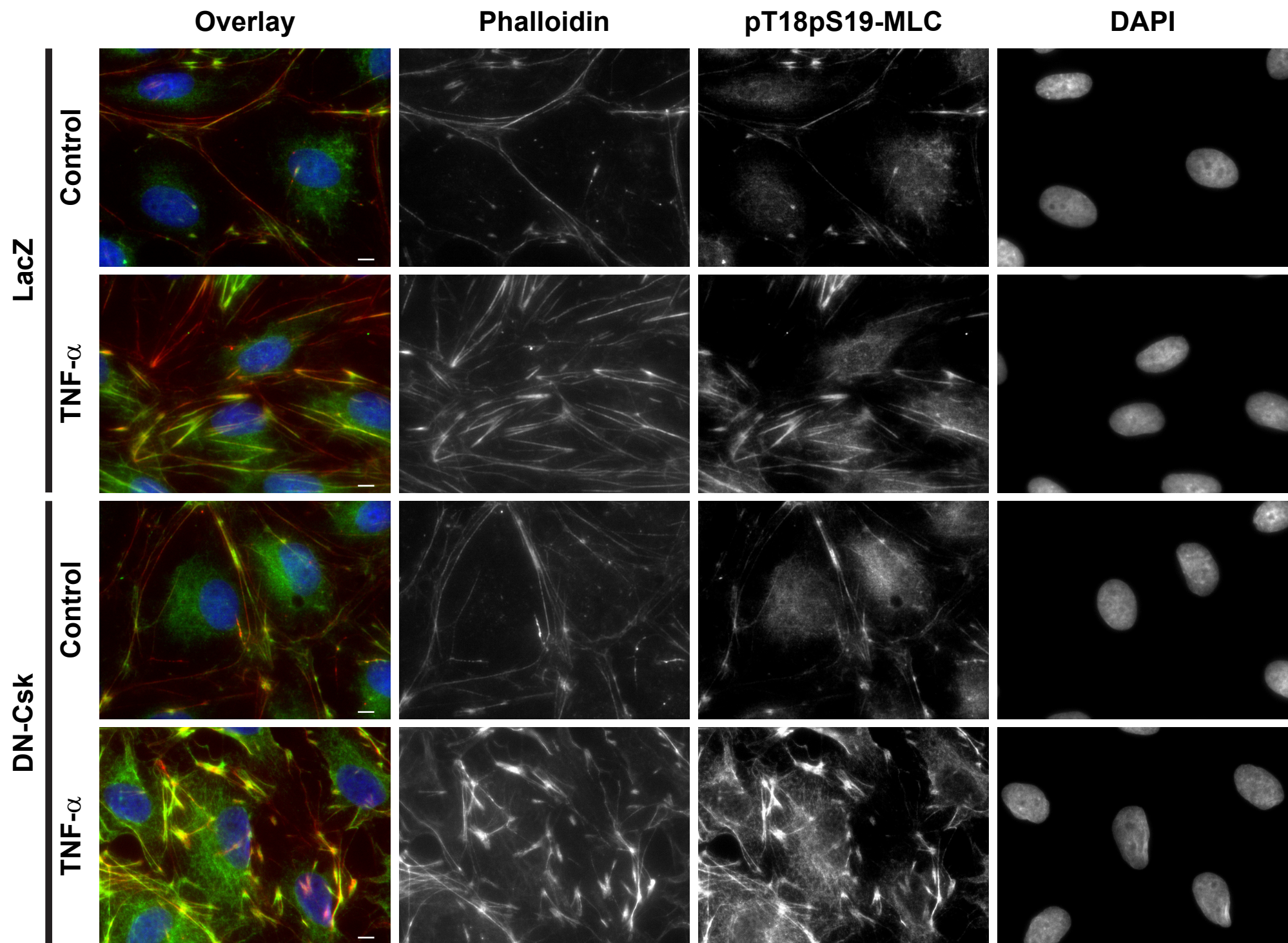

S3 Fig

Supplement: S3 Fig — Cells were seeded at confluence and incubated for 72 hours. Then, growth media was replaced by low serum media and cells were infected with adenovirus to express either LacZ (control) or DN-Csk. After another 16 h, cells were treated with low-dose TNF-α. Cells were fixed at 24 h post TNF treatment and immunofluorescence microscopy was performed to detect phosphorylated (pT18pS19) myosin light chain and F-actin (phalloidin). Nuclei were counterstained with DAPI. Note the strong colocalization of F-actin bundles and phosphorylated MLC in LD-TNF/DN-Csk cells. Results are representative of at least three independent experiments. (PDF) [file pone.0161975.s003.pdf]

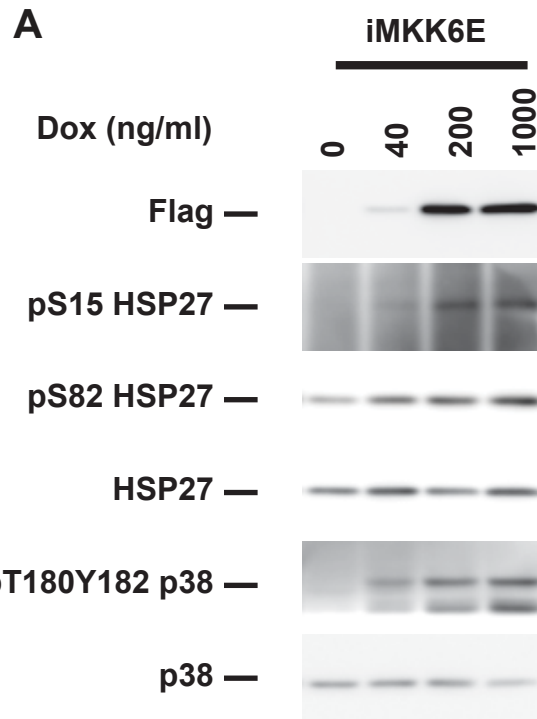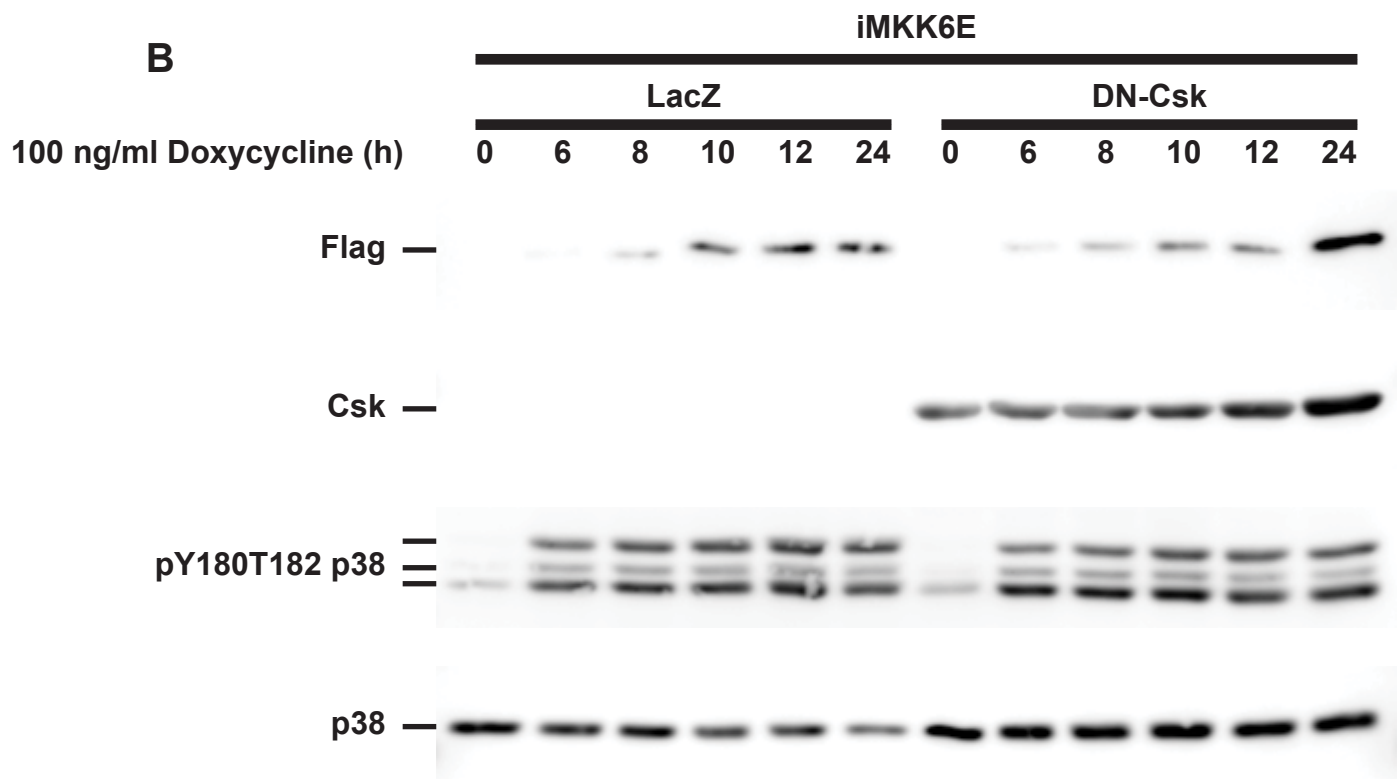

Supplement: S4 Fig — A, Pre-confluent monolayers of HDMEC were infected with lentivirus containing active FLAG-tagged MKK6 construct (iMKK6E) under the control of an inducible promoter. These cells were reseeded and allowed to become confluent for 3 days. Then, confluent iMKK6E-HDMEC monolayers were treated with varying concentrations of doxycycline to induce the expression of MKK6E in low serum media. Cells were lysed 24 h after doxycycline addition and blotted to detect the FLAG tag, as well as phosphorylated and total p38 and HSP27. B, Cells were infected with 0.1 μl/ml lentivirus and cultured as in A. Then, confluent iMKK6E-HDMEC monolayers were infected with adenovirus to express either LacZ or DN-Csk. After additional 24 h of incubation, cells were treated with or without doxycycline to induce the expression of MKK6E in low serum media at different time points and lysed 24 h later. Shown are blots to detect FLAG tag, Csk and phosphorylated and total p38. (PDF) [file pone.0161975.s004.pdf]

**A**

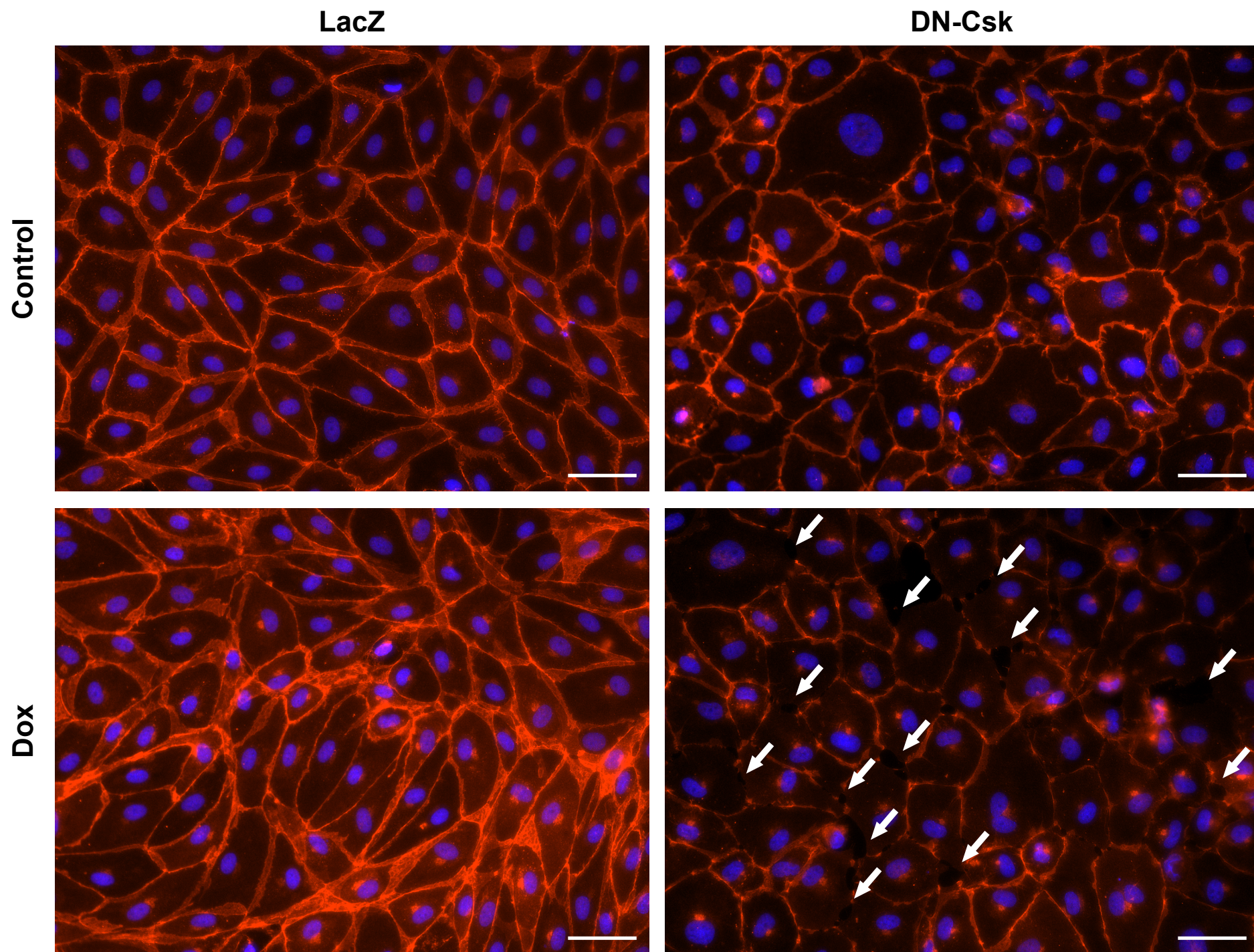

**S5 Fig**

**B****Overlay****Phalloidin****pY416 Src****DAPI****LacZ****Control**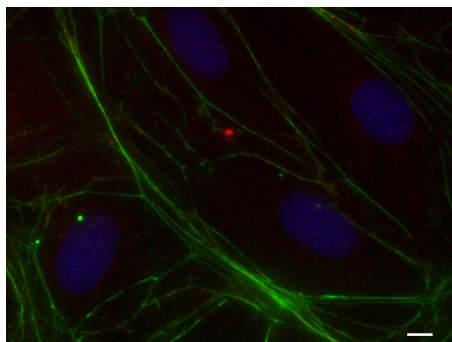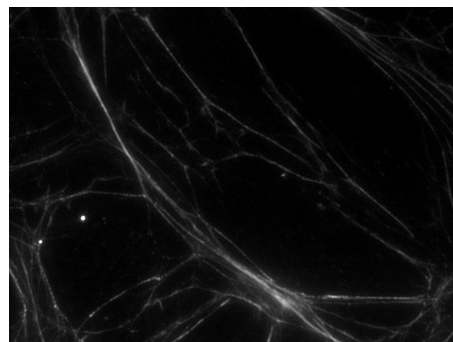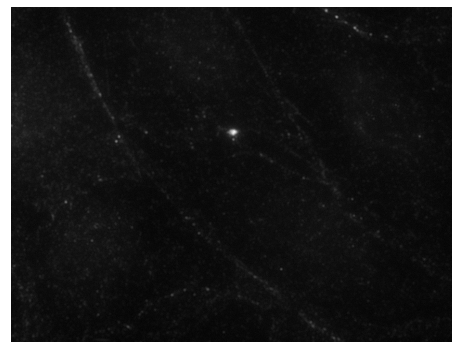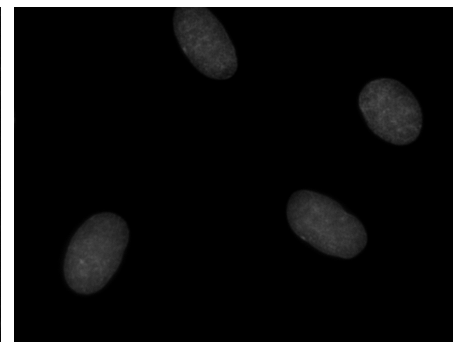**Dox**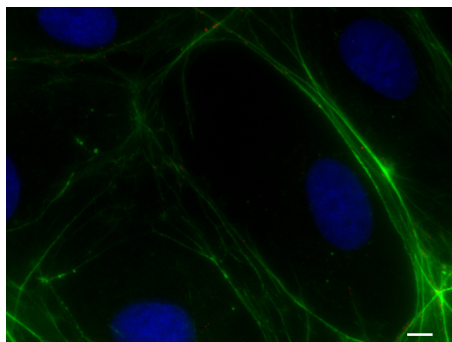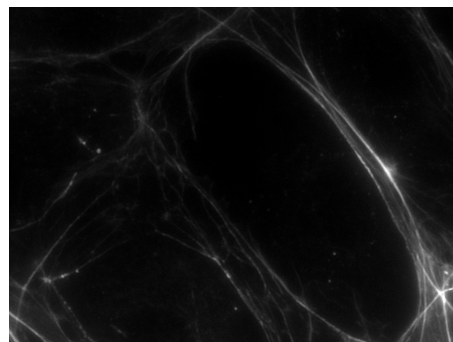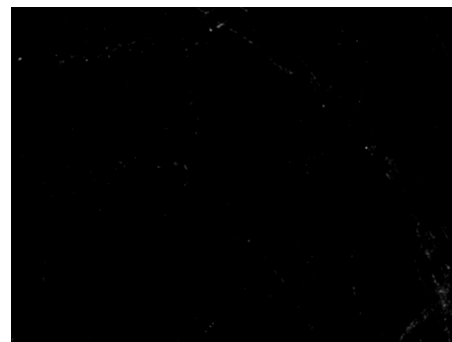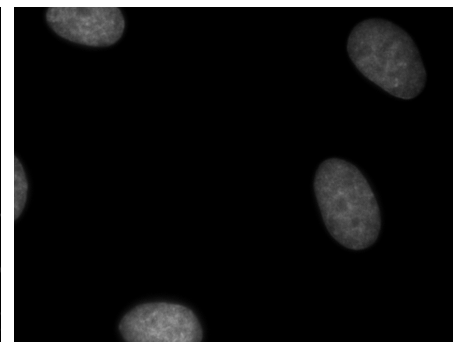**DN-Csk****Control**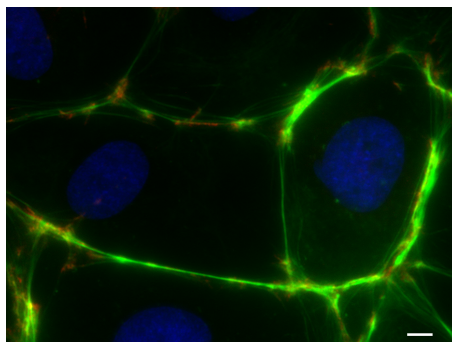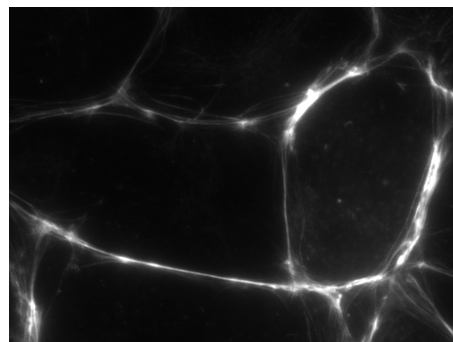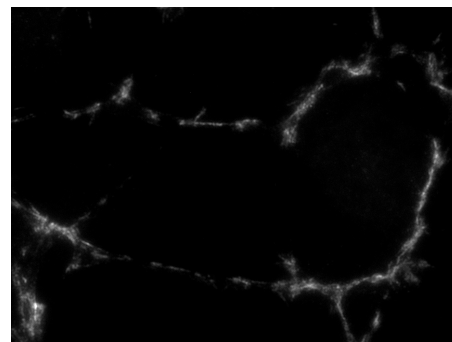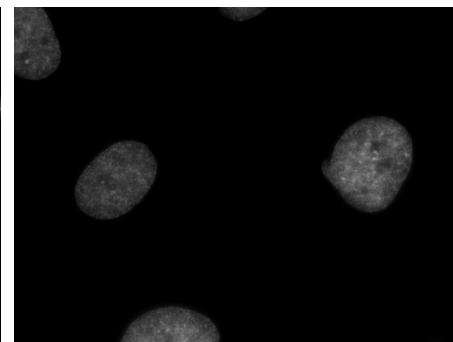**Dox**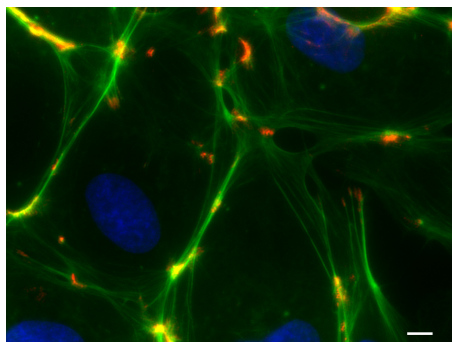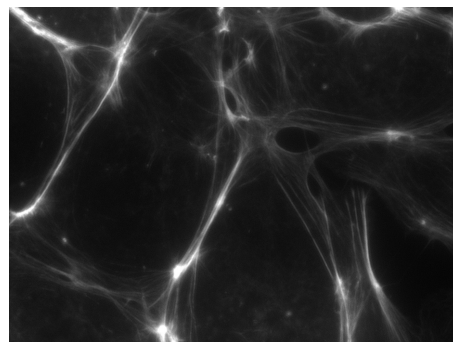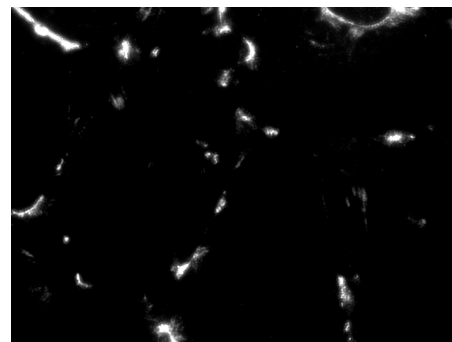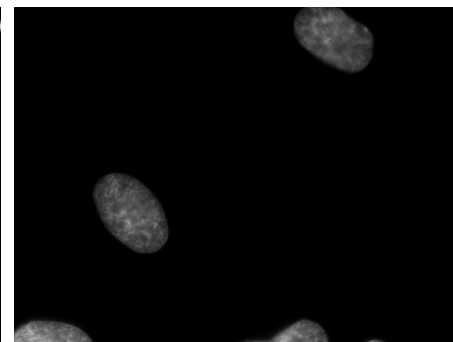

Supplement: S5 Fig — Pre-confluent monolayers of HDMEC were infected with lentivirus containing active FLAG-tagged MKK6 construct (iMKK6E) under the control of an inducible promoter. These cells were reseeded and allowed to become confluent for 3 days. Then, confluent iMKK6E-HDMEC monolayers were infected with adenovirus to express either LacZ or DN-Csk. After an additional incubation of 24 h, cells were treated with or without doxycycline to induce the expression of MKK6E in low serum media. Cells were fixed 18 h after doxycycline addition and stained for VE-cadherin (A) or active (pY416) Src and F-actin (phalloidin) (B). Nuclei were counterstained with DAPI. Note the lack of radial stress fibers or actin bundles in all four conditions. Results are representative of three independent experiments. Bars: 50 μm (A) and 5 μm (B). (PDF) [file pone.0161975.s005.pdf]

**A**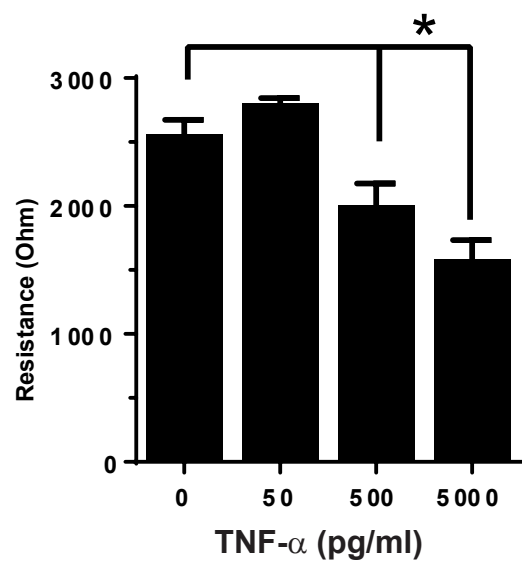**B**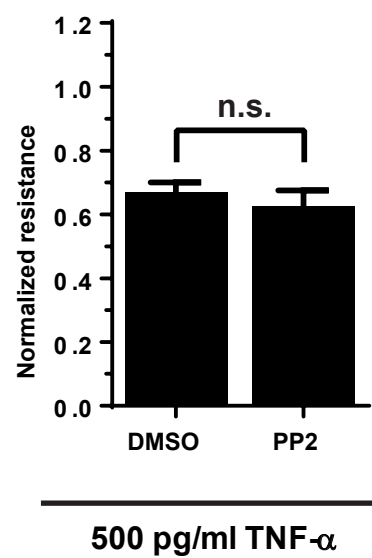**C**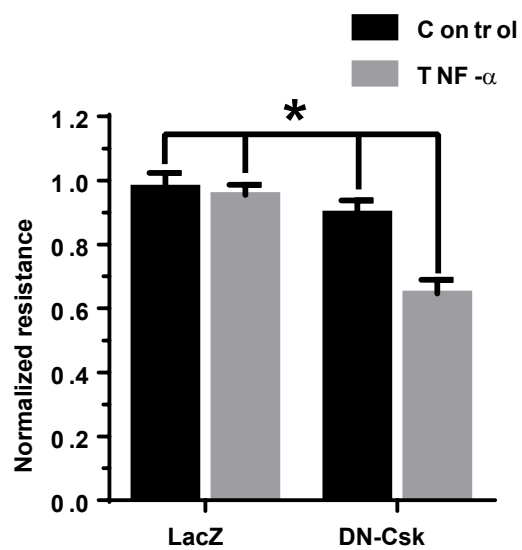**D**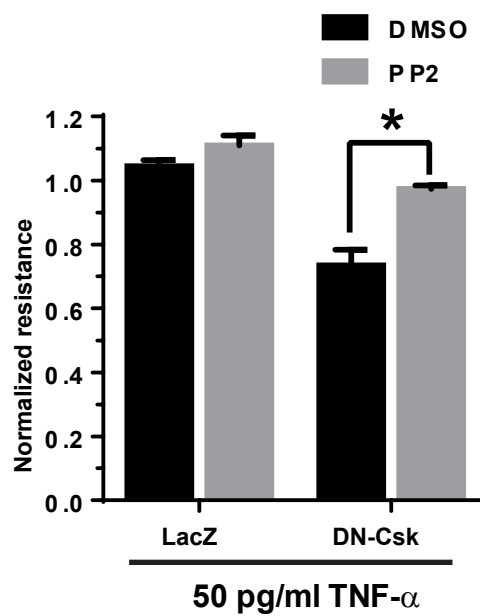

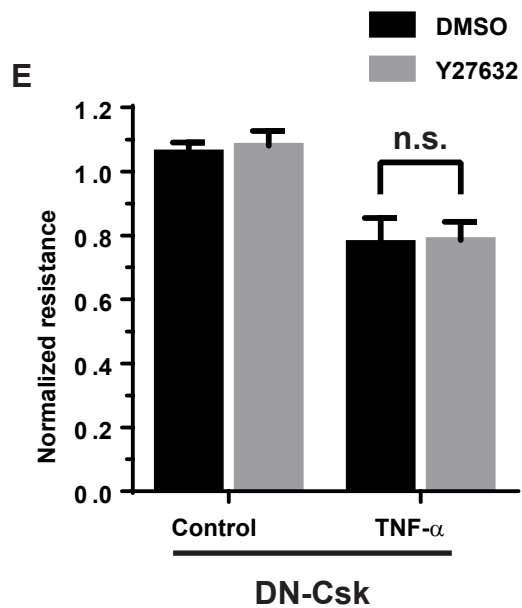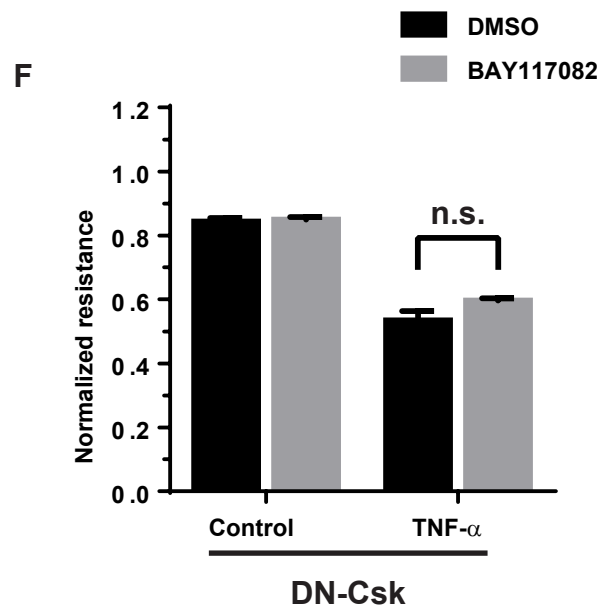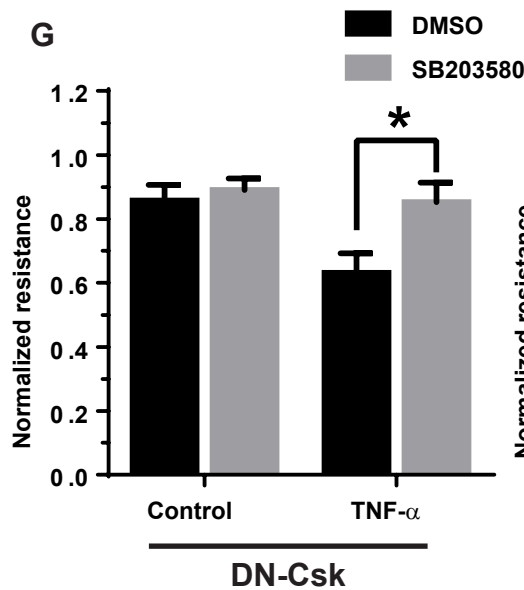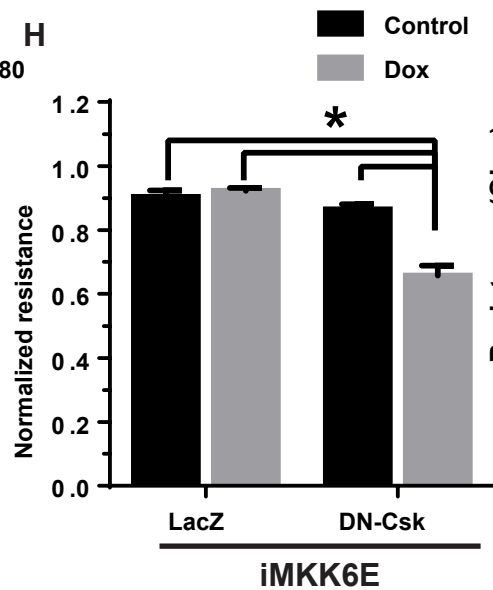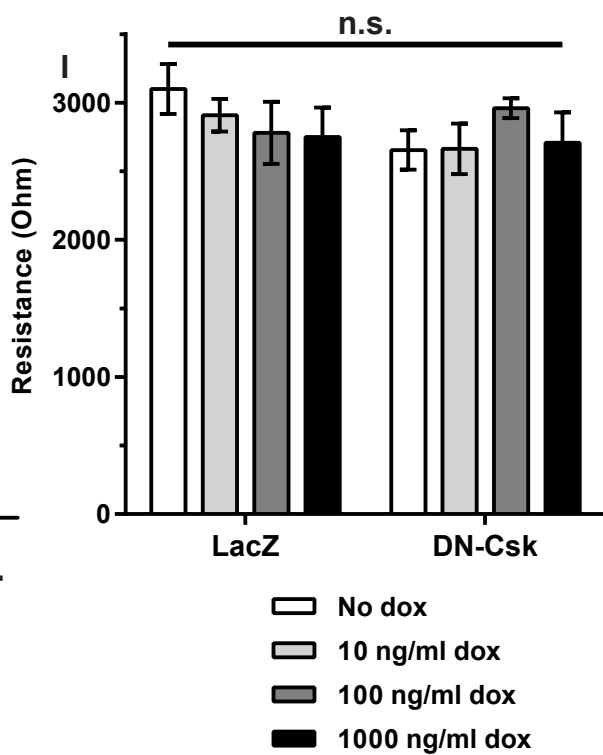

Supplement: S6 Fig — Data presented as mean±SEM of at least three independent experiments combined. Panels A-H correspond to Figs 1A, 1B, 2B, 2D, 4A, 5B, 6C and 7C respectively. A, One-way ANOVA with Dunnett post-hoc comparison against control. B, Unpaired T test of TNF-α-treated cells in the presence of either DMSO or PP2. C-H, Two-way ANOVA and Tukey’s multiple comparison post-hoc test. Asterisks denote p<0.05, while n.s. denote a non-significant change (p>0.05). (PDF) [file pone.0161975.s006.pdf]
